# Decellularized biological matrices for the repair of rotator cuff lesions: a systematic review of preclinical *in vivo* studies

**DOI:** 10.3389/fbioe.2024.1345343

**Published:** 2024-02-01

**Authors:** Giorgia Codispoti, Melania Carniato, Silvia Brogini, Alessia Romanelli, Lucia Martini, Gianluca Giavaresi, Matilde Tschon

**Affiliations:** Surgical Sciences and Technologies, IRCCS Istituto Ortopedico Rizzoli, Bologna, Italy

**Keywords:** animal models, decellularized biological patches, efficacy, rotator cuff lesions, systematic review

## Abstract

**Background:** Rotator cuff tears (RCTs), resulting from degeneration or trauma of the shoulder tendons, are one of the main causes of shoulder pain. In particular, massive RCTs represent 40% of all injuries, require surgical treatment, and are characterized by poor clinical outcomes and a high rate of failure. In recent years, the use of biological decellularized patches for augmentation procedures has received great interest owing to their excellent self-integration properties, improving healing and, thus, presenting an innovative therapeutic option. However, the findings from clinical studies have emerged with conflicting viewpoints regarding the benefits of this procedure, as an excessive tension load might compromise the integrity of the tendon-to-bone connection when the patch exhibits low elasticity or insufficient strength. This could prevent the healing process, leading to unpredictable results in clinical practice.

**Methods:** This systematic review was conducted following Preferred Reporting Items for Systematic reviews and Meta-Analyses (PRISMA) guidelines across three databases (PubMed, Scopus, and Web of Knowledge) to underline the results obtained in preclinical studies involving animal models of RCT surgeries that utilized the biological decellularized matrix augmentation technique in the last 5 years.

**Results:** Thirteen articles were included after the screening, and the SYRCLE tools were applied to assess the risk of bias in *in vivo* studies. Open-surgery techniques were conducted to create tendon defects or detachment in different animal models: rat (31%), rabbit (46%), dog (15%), and sheep (8%). Patches decellularized with non-standardized protocols were used in 77% of studies, while commercially available matrices were used in 15%. Of the studies, 31% used allogenic patches, 61% used xenogenic patches, and 8% utilized both xenogenic and autologous patches.

**Conclusion:** Overall, this review provides a comprehensive overview of the use of acellular patches and their effective therapeutic potential in rotator cuff (RC) repair at the preclinical level with the aim of expanding the strategies and matrices available for surgeons.

**Systematic review registration:** https://www.crd.york.ac.uk/prospero/, identifier CRD42023468716.

## 1 Introduction

Rotator cuff disorders include a wide spectrum of pathologies related to the different anatomical structures that make up the rotator cuff, and their incidence increases with age. In humans, the rotator cuff consists of the subscapular, teres minor, supraspinatus, and infraspinatus muscles and their tendons at the neck of the humeral head, which provide dynamic stabilization of the glenohumeral joint and, thus, shoulder motion. Rotator cuff injuries are usually degenerative conditions due to trauma or degeneration of the shoulder tendons and are the most common cause of pain and fatigue (Zhou et al., 2023). Rotator cuff tears (RCTs), which include partial or full-thickness injuries, can range in size from small to massive; full-thickness injuries are also classified by tear size into small (<1 cm in length), medium (from 1 to 3 cm), large (from 3 to 5 cm), and massive (greater than 5 cm) tears and by Patte or modified Patte classifications according to MRI measurements (tendon retraction in the frontal plane, supraspinatus atrophy, and supraspinatus muscle fat infiltration) (Lippe et al., 2012; Dang and Davies, 2018). The modified Patte classification can predict both the risk of re-tear after surgery and tendon irreparability (Guo et al., 2020). Despite recent advancements in surgical techniques, fixation biomaterials, and rehabilitation programs, massive RCTs represent 40% of all injuries and often have a poor clinical outcome and a high rate of failure compared to smaller RCTs (Di Benedetto et al., 2021; Yang et al., 2022). A successful outcome for small- and medium-size partial or full-thickness tears is obtained by non-surgical or conservative options, such as periscapular or deltoid musculature strengthening and functional rehabilitation to increase the range of joint motion and restore muscle strength and joint coordination (Kuhn et al., 2013). In addition to physiotherapy, glucocorticoid injections can be used to reduce inflammation and pain although they can cause spontaneous tendon rupture and slow healing (Dang and Davies, 2018; Zhou et al., 2023). Surgical treatment is considered based on factors such as the size, thickness, and muscle quality of the tear. It is the preferred option for lesions that cannot be treated with conservative options, particularly in young patients. Various surgical strategies have been proposed for the treatment of massive tears, including arthroscopic debridement followed by biceps tenotomy or tenodesis and subacromial decompression, complete or partial tear repair, tendon transfer, arthroscopic superior capsular reconstruction, and total arthroplasty. Advances in surgical techniques for the repair of RCTs have made it possible to achieve the goals of reducing pain, restoring function and motion, restoring the biomechanical properties of the rotator cuff, and promoting healing. Nevertheless, surgical treatment of massive RCTs has a high failure rate (up to and over 90% of cases (McElvany et al., 2015)) due to fat infiltration, tension on the repaired site, reduction of the acromion humeral distance, and patient characteristics, as reported in many studies (Di Benedetto et al., 2021; Zhou et al., 2023). An alternative surgical technique for the repair of RCTs is patch augmentation, which can improve the strength of the tendon–bone junction and the healing and self-integration processes due to its ability to promote vascularization and cellular growth (Cai et al., 2018). In addition, patch augmentation may reduce the re-tear rate compared to surgical partial repair of massive RCTs with low-grade fat infiltration and pain scores (Mori et al., 2013; de Andrade et al., 2022). The overlap of tendon and bone by the patch is performed by open surgical techniques or arthroscopically. Patch augmentation can be biological (animal or human, such as extracellular matrix-based patches), synthetic, or biosynthetic (degradable or non-degradable) (Veronesi et al., 2020). Synthetic patches have shown good results, but they can induce an immune response as a foreign body reaction; they do not have the same mechanical properties as native tissue and, unlike biological patches, can negatively influence healing due to the stress shielding phenomenon. Instead, biological matrices can mimic the extracellular matrix microenvironment and, thus, promote cellular differentiation, growth, and tissue repair. However, the results of clinical studies have highlighted conflicting views on the benefits of this procedure, as tension overload may damage the tendon–bone interface if the patch has low elasticity or is too weak, and the healing process may be inadequate, leading to unpredictable clinical outcomes (Avanzi et al., 2019; Cobb et al., 2022).

From a translational perspective, using animal models to evaluate the efficacy of decellularized patches can provide important information on safety and efficacy although they do not provide a perfect representation of the clinical condition (Yang et al., 2022). For these reasons, the aim of this systematic review is to highlight the benefits obtained in preclinical studies on animal models of RCT surgery using the biological decellularized matrix augmentation technique to promote healing and reduce the rate of re-tears after surgery.

## 2 Materials and methods

### 2.1 Search strategy

The present literature review involved a systematic search carried out according to the PRISMA statement in three electronic databases (PubMed, Scopus, and Web of Knowledge: www.pubmed.gov, www.scopus.com, and www.webofknowledge.com). The search was performed using the following keywords: “(acellular OR decellularized) AND (dermis OR graft OR dermal matrix OR scaffold OR patch OR biomaterial OR membrane) AND (shoulder OR rotator cuff).” The search was limited to papers published in the period from 1 January 2018 to 31 December 2023 and written in English.

The screening process and analysis were conducted separately by three independent observers (SB, GC, and MT) using the collaboration platform Rayyan (Ouzzani et al., 2016). First, the articles were screened based on title and abstract using the following inclusion criteria: papers investigating the efficacy of decellularized matrices from different sources for treating rotator cuff tears and using preclinical *in vivo* models. The exclusion criteria encompassed articles written in other languages, reviews, unavailable abstracts or full texts, editorials, technical notes or conference proceedings, *in vitro* and clinical studies, and publications lacking animal models, rotator cuff lesions, and decellularized patches. The reference lists of the included papers were screened to obtain further studies. Disagreements were resolved by discussion, and where resolution was not possible, the fourth and fifth reviewers were consulted (MC and LM).

The protocol was registered at inception in the PROSPERO register (record no. CRD42023468716).

### 2.2 Data extraction

The papers’ main characteristics were extracted by GC and MT based on the animal model, species, strain, number, sex, lesion’s site and dimensions, surgical procedure and treatments, decellularized patch used with the source and tissue, decellularization protocol, main tests with selected experimental times, main findings, and the first author’s name with the year of publication. Data were checked for accuracy and completeness by a third author (S.B.), and disagreements were resolved by discussion, and where resolution was not possible, the fourth and fifth reviewers were consulted (M.C. and L.M.). The included papers were grouped according to the animal species.

### 2.3 Risk of bias assessment

A quality assessment of the *in vivo* studies was performed using the SYRCLE tool for animals, which comprises a 10-item checklist (Hooijmans et al., 2014). A low, high, or unclear risk of bias was scored if items were reported, not reported, or unclearly reported, respectively. The assessment was performed by two independent authors (GC and SB). Any disagreement was resolved by consensus with a third reviewer (MT).

## 3 Results

### 3.1 Search strategy

**FIGURE 1:**
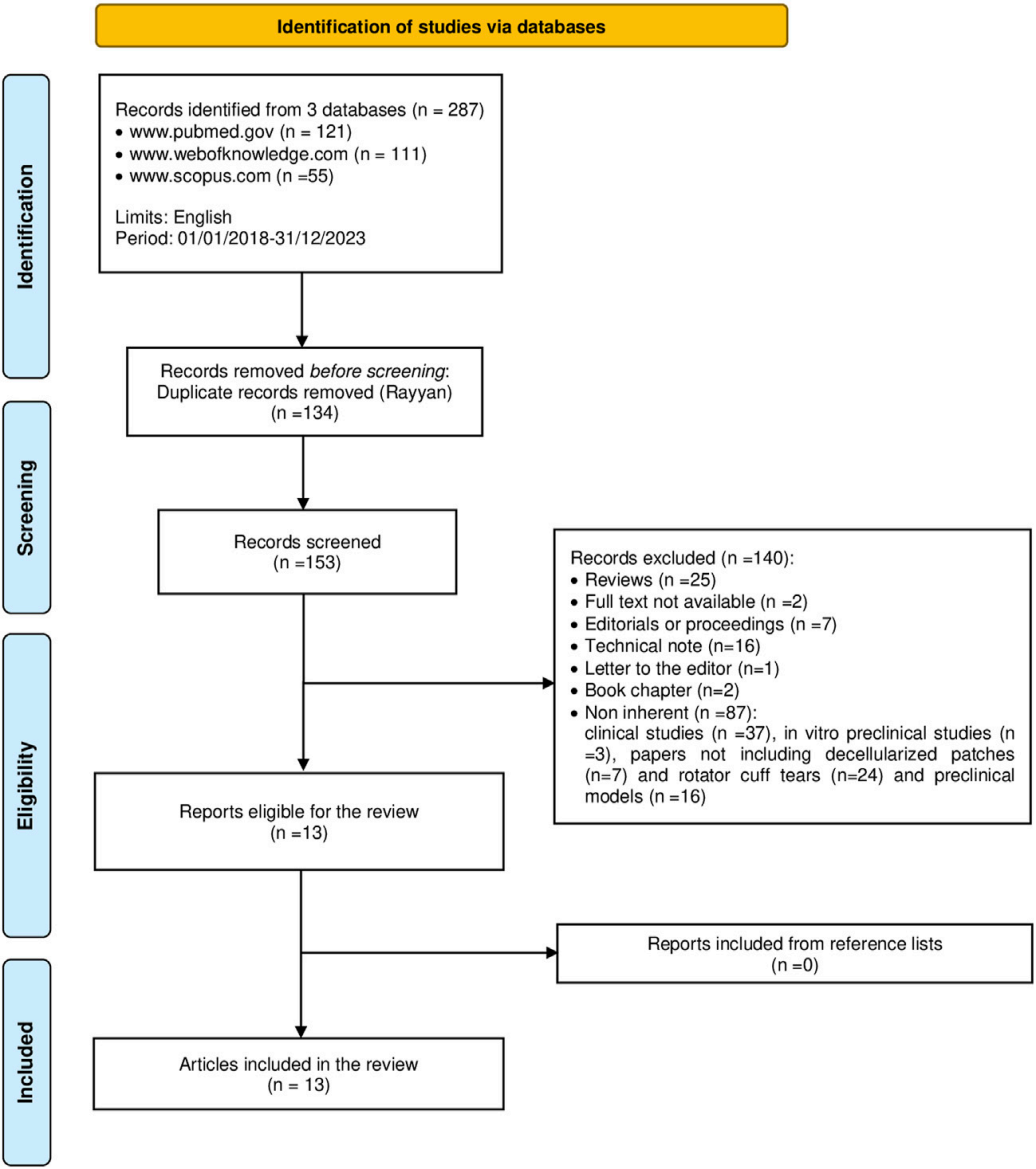
Search strategy according to the PRISMA guidelines.

The initial literature search using the above keywords was conducted according to the Preferred Reporting Items for Systematic reviews and Meta-Analyses (PRISMA) statement and yielded the following results: 121 articles were retrieved from PubMed (www.pubmed.gov), 111 from Web of Knowledge (www.webofknowledge.com), and 55 from Scopus (www.scopus.com). The resulting references were uploaded to the Rayyan platform, where each author blindly evaluated the inclusion/exclusion criteria. Duplicate articles were then identified and removed (*n* = 134). Excluded articles were reviews (*n* = 25), articles without full text (*n* = 2), editorial comment or proceedings (*n* = 7), technical notes (*n* = 16), letters to the editor (*n* = 1), book chapter (*n* = 2), clinical trials (*n* = 37), *in vitro* preclinical studies (*n* = 3), and articles not including decellularized patches (*n* = 7), preclinical models (*n* = 16), or rotator cuff tears (*n* = 24). A list of excluded articles is provided in Supplementary Table S1. No articles were retrieved from the reference lists. Finally, 13 *in vivo* preclinical studies were included in this systematic review (Figure 1). The included papers were grouped and discussed according to animal species.

### 3.2 Animal species

Data were extracted per animal species: rats are reported in Table 1, rabbits in Table 2, sheep in Table 3, and dogs in Table 4. Most of the studies used rabbits (6/13 papers, 46%), followed by rats (4/13, 31%), dogs (2/13, 15%), and sheep (1/13, 8%).

**TABLE 1:** Summary of rat preclinical studies.

Animal model: strain, number, sex, and groups, if any	Type of lesion (site and dimension)	Surgical procedure and treatments	Decellularized patch (source and tissue)	Decellularization protocol	Main tests	Experimental times	Main results	Reference
18 female Wistar rats, 6 per group:	Unilateral detachment (SST, n.r.)	Augmentation after 3 weeks with or without scaffolds	Rat cortical DBM vs*.* commercially available human dermal matrix (GraftJacket™)	Registered trademark	Macroscopic analyses: continuity between the repaired tendon and bone;	Macroscopic analyses, histology, and pqCT: 9 weeks from lesion induction	Macroscopic analyses: in the DBM group, the scaffold could not be discerned from the other tissues similar to the MSCs-only group, while in the GraftJacket™ group, it was visible;	Thangarajah et al. (2018)
1) DBM, 1.5 cm x 3/5 cm + 1 × 10^6^ MSCs;					histology: the modified Movin scale for tendon degeneration assessment; Ide *et al.*’s scoring system for enthesis’ maturation evaluation;		histology:	
2) human dermal matrix, 1.5 cm x 3/5 cm + 1 × 10^6^ MSCs;							↑ enthesis score in DBM compared to the dermal matrix group;	
3) only MSCs (without scaffold) + 1 female Wistar rat for MSCs					pqCT: BMD		pqCT: ↓ BMD in all groups	
18 male Sprague–Dawley rats, 6 per group:	Bilateral tear at the bone-to-tendon insertion (SST, n.r.)	Repair with or without patch (loaded or not with MSC)	Bovine pericardial patch	n.r.	MRI: evaluation of tendons’ and patches’ conditions for biomechanical analyses;	MRI: at 4 weeks after repair;	MRI: no differences;	Shim et al. (2022)
1) without any patch;					macroscopic observations and histology: evaluation of repair to bone–tendon insertion;	macroscopic analyses, histology, and biomechanical tests: at 8 weeks after repair;	Macroscopic observations, histological evaluation, and histomorphometry: no re-tears except in 1 ctr shoulder and patch (without MSC) groups; native tendon tissue and patch not visible; denser and more aligned collagen tissue in the patch + MSC group;	
2) pericardial patch, 10 mm length x 5 mm + rat MSCs;					histomorphometry: the modified scoring system at the bone-to-tendon interface, collagen maturity and fiber orientation and density, and vascular formation;	histomorphometry: at 2 and 4 weeks after repair	biomechanical tests:	
3) pericardial patch, 10 mm length x 5 mm without rat MSCs					biomechanical tests: uniaxial tensile stress tests		↑ load-to-failure in patch and patch + MSCs groups	
Male Sprague–Dawley rats: sheets, 3 mm × 2.5 mm × 0.25 mm, of acellular rabbit fibrocartilage with loaded with and without C-SDF and CXCR4+ SMSCs	Unilateral detachment (SST, n.r.)	Augmentation	Rabbit fibrocartilage from pubic symphysis	Cycles of freeze/thaw, SDS, Triton X-100, RNase, and DNase and freeze-dried	SMSC labeling and tracking;	SMSC labeling and tracking: on days 0, 4 and 14;	*In vivo* tracking showed the presence of SMSCs in the lesion site up to 14 days;	Chen et al. (2020)
					micro-CT: BV/TV, BMD, and Tb.Th measurement;	micro-CT, histology, and histomorphometry: at postoperative 1 and 2 months;	micro-CT, histology, and histomorphometry:	
					histology and histomorphometry: application of healing fibrocartilage score;	biomechanical tests: at postoperative 2 months	↑ BV/TV and Tb.Th than controls of disorganized woven bone at 4 weeks;	
					biomechanical tests: failure load and stiffness analyses		↑ BV/TV, BMD, Tb.Th, and healing scores of mature bone in the scaffold loaded with CXCR4+ SMSCs and C-SDF-1α/CXCR4+SMSCs groups at 8 weeks;	
							biomechanical tests: ↑ failure load and stiffness	
12 male Sprague–Dawley rats, 6 per group:	Bilateral defects (SST, 3 × 5 mm)	Augmentation	Human dermal matrix patch (GraftJacket™)	Registered trademark	Histology and histomorphometry: N. of chondrocytes, % of aligned chondrocytes, area of fibrocartilage, and non-collagen/collagen ratio;	Histology, histomorphometry, immunohistochemistry, and biomechanical tests: at 12 months after the procedure	Histology, histomorphometry, and biomechanical tests:	Ide and Tokunaga (2018)
1) human dermal matrix, 0.6-mm-thick;					immunohistochemistry staining for *COL2* and *COL3*;		↓ all histomorphometric and biomechanical parameters;	
2) unoperated					biomechanical tests: ultimate load-to-failure, stiffness, cross-sectional area, and ultimate stress-to-failure analyses		immunohistochemistry: the tendon–bone interface was immunostained only for collagen type 1	

**TABLE 2:** Summary of rabbit preclinical studies.

Animal model: strain, number, sex, and groups, if any	Type of lesion (site and dimension)	Surgical procedures and treatments	Decellularized patch (source and tissue)	Decellularization protocol	Main tests	Experimental times	Main results	Reference
8 male New Zealand rabbits:	Bilateral lesions (subscapularis tendons, 5 mm in length)	Repair with DTS and contralateral side used as ctr	Allograft DTS (rabbit gastrocnemius tendons)	Gastrocnemius muscle tendon decellularized with chemical (SDS and Triton X-100) and enzymatic agents (EDTA and aprotinin)	Macroscopic analyses: assessment of the integration between the DTS and tendon and the inflammatory reaction;	Macroscopic analyses and histology: 2 and 8 weeks	Macroscopic analyses:	de Lima Santos et al. (2020)
1) DTS;					histology: evaluation of cellular infiltration, inflammatory response, and collagen arrangement		↑connective tissue and inflammatory reaction at 8 weeks compared to 2 weeks;	
2) untreated							histology:	
							↑cell infiltration after 8 weeks compared to 2 weeks	
8 male New Zealand rabbits:	Bilateral lesions (subscapularis tendons, 5 mm in length)	Repair with DTS and contralateral side used as ctr	Allograft DTS (rabbit gastrocnemius tendons)	Gastrocnemius muscle tendon decellularized with chemical (SDS and Triton X-100) and enzymatic agents (EDTA and aprotinin)	Macroscopic analysis: evaluation of DTS integration with RC;	Macroscopic analyses and histology: 2 and 8 weeks	Macroscopic analysis: DTS was in progressive integration with RC;	de Lima Santos et al. (2020)
1) DTS;					histology: semi-quantitative and automated analysis of nuclear material removal for cell infiltration measurement		histology:	
2) untreated							↑cell infiltration after 8 weeks compared to 2 weeks;	
							↑ area occupied by nuclear structures after 2 and 8 weeks	
24 male New Zealand rabbits, 6 per group:	Bilateral detachment (IST, n.r.)	Repaired with or without scaffolds	DCB-ECM (n.r.)	Physical and chemical methods using a defatting solvent, freeze-drying, and demineralizing in a solution with 8% LiCl_2_, 6.5% formic acid, and 0.6 N hydrochloric acid	Macroscopic analysis;	Macroscopic analysis: 2 and 12 weeks;	Macroscopic analysis: fibrosis reaction found at the repaired sites;	He et al. (2021)
1) Ctr;					immunofluorescence staining: identification of stromal cells;	immunofluorescence staining: 2 weeks after surgery;	immunofluorescence staining:	
2) DCB;					micro-CT: BV/TV ratio and Tb.Th;	micro-CT, histology, and biomechanical tests: 12 weeks after surgery	↑stromal cells in hDCB-ECM than in hDCB, DCB, and ctr groups;	
3) hDCB,					histology and biomechanical tests: new fibrocartilage tissue formation and cross-sectional area of IST; ultimate tensile stress; and Young’s modulus		↑ area of positive staining of BMP-2 in the hDCB-ECM group;	
4) hDCB-ECM							micro-CT:	
							↑ BV/TV ratio and Tb.Th in the hDCB-ECM group;	
							histology and biomechanical tests:	
							↑ new fibrocartilage in the hDCB-ECM group; no significant difference in the cross-sectional area;	
							↑ ultimate tensile stress and Young’s modulus in the hDCB-ECM group	
16 New Zealand rabbits:	Bilaterally chronic retracted RC tear (n.r.)	SCR realized 8 weeks after surgically induced tear	Xenograft HDG vs*.* autologous TFL	n.r.	Macroscopic analysis: healing rate;	Macroscopic analysis, histology, and biomechanical testing: at 12 weeks	Macroscopic analysis: complete healing in all samples of both treated groups;	Yildiz et al. (2019)
1) nine per treatment group:					histology: enthesis maturation score and graft-to-bone healing;		histology: no differences in the enthesis maturation scores;	
→ HDG, 20 mmx 10 mm in width x 1.26–1.75 mm in thickness, right shoulders					biomechanical testing: loading tests and tensile strength		↑collagen fiber density in the TFL group; delayed healing and inflammatory response in the HDG group;	
→ TFL autograft, 1.26–1.75 mm in thickness, left shoulders							Biomechanical testing: all the specimens passed cyclical loading tests and did not pass the load-to-failure tests	
2) 7 ctr group								
54 White New Zealand rabbits treated with or without DUCWJ, 2 cm × 1 cm x 2 mm, scaffold	Unilateral defects (SST, 5 mm in length)	Bridging	DUCWJ Scaffold	Freeze–thawing five times and decellularization in SDS, followed by Triton X-100, RNase, and DNase	Macroscopic observations: evaluation of RCT repair;	Macroscopic, histology, RT-PCR, and biomechanics analyses: at 4, 8, and 12 weeks	Macroscopic: repaired tendons in continuity with bone in the DUCWJ group;	Yuan et al. (2022a)
					histology and immunohistochemistry: tendon scoring system by Suh et al;		histology and immunohistochemistry: ↑ number and diameter of collagen fibers in the DUCWJ group at 12 weeks;	
					immunohistochemical staining for *COL1A1* and *COL3A1*;			
					RT-PCR: *COL1A1*, *COL3A1*, *TNC*, and *TNMD*;		↓ inflammation and vascularity in the DUCWJ group;	
					biomechanical analyses: tensile strength		↑ *COL1A1*	
							↓ *COL3A1* at 8 and 12 weeks.	
							↓ scores in the DUCWJ group;	
							RT-PCR:	
							↑ *COLA1*, *TNC*, and *TNMD* and ↓ *COL3A1* in the DUCWJ group; biomechanical tests:	
							↑ maximum failure load and tensile modulus at 4, 8, and 12 weeks in the DUCWJ group	
45 New Zealand rabbits: repair with patches, 2 cm × 1 cm x 2 mm, conjugated with and without KGN or left untreated, as ctr	Bilateral defects (IST, 5 mm in length)	Augmentation	Human umbilical cord Wharton’s jelly with and without KGN conjugation	Cycles of freeze/thaw, SDS, Triton X-100, RNase, and DNase	Macroscopic analyses: RC examination;	Macroscopic analyses, histology, histomorphometry, immunohistochemistry, RT- PCR, and biomechanical tests: at 1, 2, and 3 months	Macroscopic analyses: no adverse reactions;	Yuan et al. (2022b)
					histology and histomorphometry: scoring system for tendon–bone repair;		histology and histomorphometry: ↑ healing score, collagen deposition, neo-fibrocartilage formation, and fiber organization in patch-treated groups;	
					immunohistochemistry staining for *COL2*;			
					RT-PCR: *COL1*, *COL2*, *ACAN*, and *TNC*;		RT-PCR: ↑ *COL1A1*, *COL2*, *ACAN*, and *TNC* at 4, 8, and 12 weeks;	
					biomechanical tests: ultimate load-to-failure and tensile modulus analyses			
							biochemical tests: ↑ maximum load-to-failure and stiffness	

**TABLE 3:** Summary of sheep preclinical studies.

Animal model: strain, number, sex, and groups, if any	Type of lesion (site and dimension)	Surgical procedures and treatments	Decellularized patch (source and tissue)	Decellularization protocol	Main tests	Experimental times	Main results	Reference
30 Rambouillet cross sheep with or without biphasic cancellous allograft after 6 weeks from surgery	Full-thickness RCTs (IST, n.r.)	RCR	Biphasic interpositional cancellous allograft	n.r.	Macroscopic analysis: scar tissue and amount of tendon retraction and muscle atrophy;	Macroscopic analysis, histology, and histomorphometry: after 3, 6, and 12 weeks	Macroscopic analysis: all IST thickened and covered with fibrotic scar tissue;	Credille et al. (2023)
					histology and histomorphometry: inflammatory cell infiltrates, signs of implant degradation, particulate debris, collagen arrangement, cellularity, neovascularization, enthesis, and the presence of Sharpey-like fibers		histology and histomorphometry: ↓ scores at 6- and 12-week time; progressive collagen arrangement both in treated and control groups; initial tendon healing at 3 and 6 weeks and more mature fibrocartilaginous enthesis at 12 weeks	

**TABLE 4:** Summary of dog preclinical studies.

Animal model: strain, number, sex, and groups, if any	Type of lesion (site and dimension)	Surgical procedures and treatments	Decellularized patch (source and tissue)	Decellularization protocol	Main tests	Experimental times	Main results	Reference
16 purpose-bred:	Bilateral half-thickness lesions (SST, 6.5–8.5 mm)	Augmentation	AM (human amnion), AF (human dermis), or bovine RMP (n.r.)	n.r.	Clinical evaluation: lameness grade, forelimb function at a trot, CROM, and VAS pain scores;	Clinical evaluation: before surgery and at 3 and/or 6 months;	Clinical evaluation:↓ CROM in all groups↑ VAS in the DB and RMP group;	Smith et al. (2020)
1) DB;					ultrasonography: SST appearance and integrity and the presence of biceps impingement;	ultrasonography: before surgery and at 6 weeks, 3 months, and/or 6 months;		
2) AM;					MRI: bone marrow lesions, tendon—bone junction, tendon, and muscle;	MRI, arthroscopic assessment, macroscopic examination, biomechanical testing, and histology: at 3-and 6 months		
3) AF;					arthroscopy: articular cartilage, biceps tendon impingement, synovium, SST, and lateral glenohumeral ligament;		ultrasonography: at 6 weeks and 3 months SST not fully intact in the DB group. Thickened SSTs, unorganized connective tissue, and biceps impingement in the DB group;	
4) RMP					macroscopic: SST integrity;		peritendinous fluid accumulation and hyperechoic, thickened tissues of SSTs in the RMP group;	
					biomechanical testing: tensile loading and stiffness;		↓ fiber number and thickness in AM and AF groups;	
					histology: Bonar scoring system		MRI: tissue bridging in all groups; hyper-intense peritendon, intramuscular, intra-articular fluid, and impingement in all;	
							↓ impingement of biceps in the AF group;	
							arthroscopy: severe synovitis, adhesions, fibrosis, and biceps tendon impingement in the DB group at 3 months and in AF and AM groups at 6 months;	
							macroscopic examination: bridging, hypervascularity, and inflammatory tissue in RMP;	
							biomechanical testing: no differences;	
							histology: less-severe pathology in AF than in AM, RMP, and DB groups; diffuse hypercellularity, mild fatty infiltration, synovial hyperplasia, and fibrosis, with focal necrosis	
26 male beagles with sheets of canine IST, 13 per group:	Unilateral detachment (IST, n. r.)	Augmentation	Canine IST	Cycles of freeze/thaw, SDS, Triton X-100, RNase, and DNase	MRI: Repaired tissue area;	MRI: at 1, 2, 3 months;	MRI: At 1- and 2-month edema and swelling in both groups, better regeneration in experimental group at 3 months;	Chen et al. (2022)
→ loaded with cUSCs;					macroscopic analyses: gap healing rate;	macroscopic analyses, micro-CT, histology, and biomechanical tests: at 3 months;	macroscopic analyses: no significant differences;	
→without cUSCs					micro-CT: BV/TV, TbTh, and TbN;		micro-CT:↑ newly formed bone, BV/TV, TbTh, and TbN;	
					histology: application of healing fibrocartilage score;			
					biomechanical tests: failure load and stiffness analyses		histology and biomechanical tests:	
							↑ histological scores and biomechanical values	

ACAN, aggrecan; AF, decellularized human dermal allograft; AM, amnion matrix cord scaffold; BMD, bone mineral density; BMP2, bone morphogenetic protein 2; BV/TV ratio, bone volume/total volume ratio; *COL1*, collagen type I; *COL1A1*, collagen type I; *COL3*, collagen type III; *COL3A1*, collagen type III; RT-PCR, real-time PCR; CROM, comfortable shoulder range of motion; C-SDF, recombinant C-terminus stromal cell-derived factor 1α; CTR, control; CXCR4, C-X-C motif chemokine receptor 4; DB, debridement; DBM, demineralized bone matrix; dH_2_O, distilled water; DTS, decellularized tendon scaffold; DUCWJ, decellularized umbilical cord Wharton jelly; ECM, extracellular matrix; EDTA, ethylenediaminetetraacetic acid; GFP, green fluorescence protein; hDCB, hierarchically demineralized cortical bone; HDG , acellular human dermal graft; IST , degenerative infraspinatus tendon; LiCl_2_, lithium chloride; micro-CT, micro-computed tomography; MRI, magnetic resonance imaging; MSCs, mesenchymal stem cells; PBS, phosphate-buffered saline; pqCT, peripheral quantitative-computed tomography; RC, rotator cuff; RCR, rotator cuff repair; RCT, rotator cuff tendon; RMP, rotation medical patch; SDS, sodium dodecyl sulfate; SMSCs, synovium-derived mesenchymal stem cells; SSP, supraspinatus; SST, supraspinatus tendon; Tb.Th, trabecular thickness; *COL2*, collagen type II; TbN, trabecular number; TDSCs, tendon-derived stem cells; TFL, tensor fascia lata; *TNC*, tenascin C; *TNMD*, tenomodulin; KGN, kartogenin; Triton X-100, t-octylphenoxypolyethoxyethanol; USCs, urine-derived stem cells; VAS, visual analog scale.

#### 3.2.1 Rats

Three studies out of the four used Sprague–Dawley rats (Ide and Tokunaga, 2018; Chen et al., 2020; Shim et al., 2022), and one utilized Wistar rats. Thangarajah et al. (2018) and Chen et al. (2020) produced a unilateral detachment of the supraspinatus tendon (SST), while Ide and Tokunaga (2018) created bilateral defects of the SSTs (3 × 5 mm). All research groups performed the augmentation technique for tendon-to-bone repair with or without scaffold: in two cases, the commercially available human dermal matrix GraftJacket™, alone (Ide and Tokunaga, 2018) or loaded with mesenchymal stem cells (MSCs) in fibrin glue (Thangarajah et al., 2018), has been used in the experimental group compared to the unoperated or defect-untreated control group; in one article, acellular xenogenic rabbit fibrocartilage from pubic symphysis loaded with recombinant C-terminus stromal cell-derived factor 1α (C-SDF), chemokine (C-X-C motif) receptor 4 (CXCR4+), and synovium-derived MSCs (SMSCs) has been utilized to repair SST detachment (Chen et al., 2020). Shim et al. (2022) and Thangarajah et al. (2018) repaired the SST lesion by applying a decellularized patch derived from bovine pericardium loaded with autologous MSCs.

Three studies out of the four reported the sex of animals: Thangarajah et al. (2018) used female rats, whereas Chen et al. (2020) and Ide and Tokunaga (2018) used male rats. In addition, the animal’s average age varied between 8 and 22 weeks (Ide and Tokunaga, 2018; Shim et al., 2022). Experimental times ranged between 2 weeks and 12 months.

Macroscopic analyses, radiological investigations, and biomechanical tests were performed to assess the efficacy of the tested decellularized patches. Macroscopic observations were necessary to qualitatively evaluate the tendon repair at tendon-to-bone insertion: GraftJacket™ insertion at the tendon interface was visible (Thangarajah et al., 2018) in comparison with the decellularized bovine pericardial patch, in which there was continuity between the repaired scaffold-tendon and the bone.

Thangarajah et al. (2018), Chen et al. (2020), and Shim et al. (2022) performed magnetic resonance imaging (MRI) and micro-CT to measure histomorphometric parameters, such as bone mineral density (BMD), bone volume (BV/TV), and trabecular thickness (Tb.Th). BMD was decreased in the GraftJacket™ group compared to non-operated controls (Thangarajah et al., 2018); however, there was an increase in BMD and abovementioned histomorphometric parameters at 8 weeks after the acellular rabbit fibrocartilage implantation compared to controls (Chen et al., 2020).

Finally, biomechanical analyses conducted by Chen et al. (2020) and Shim et al. (2022) showed higher load-to-failure and stiffness in experimental groups compared to untreated control groups; in contrast, these biomechanical parameters were significantly lower in treated animals compared to unoperated healthy controls (Ide and Tokunaga, 2018).

#### 3.2.2 Rabbits

All studies used New Zealand White rabbits; only three papers out of six reported the sex (male) or age (28–32 weeks old) (de Lima Santos et al., 2020; He et al., 2021; Santos et al., 2022). Most surgeries (67%) involved the creation of defects of 5 mm (de Lima Santos et al., 2020; Yuan et al., 2022a; Yuan et al., 2022b; Santos et al., 2022). Only He et al. (2021) performed a bilateral IST detachment. One group performed a two-step surgery to obtain bilateral chronic and retracted RCTs (Yildiz et al., 2019). Treatments comprised the use of decellularized patches in comparison with the contralateral side used as an untreated control. de Lima Santos et al. (2020) and Santos et al. (2022) investigated allograft decellularized tendon scaffold (DTS) from rabbit gastrocnemius muscle tendons; Yuan et al. (2022a) and Yuan et al. (2022b) repaired the created defects of SSTs and ISTs with decellularized umbilical cord Wharton jelly (DUCWJ) alone or conjugated with kartogenin (KGN) using the bridging technique and augmentation technique, respectively. He et al. (2021) used a demineralized cortical bone (hDCB) coated with the extracellular matrix (ECM) for IST detachment and rabbit tendon-derived stem cells (TDSCs) for enthesis healing; Yildiz et al. (2019) realized superior capsule reconstruction by grafting an acellular human dermal graft (HDG) in the right shoulders in comparison with an autologous tensor fascia lata (TFL) in the left shoulders.

Experimental times ranged between 2 weeks and 3 months. All studies performed macroscopic analyses for tendon repair and healing assessments; De Lima Santos et al. and Yuan et al. observed an increasing integration of DTS and DUCWJ, respectively, at each time point. Yuan et al. (2022b), Santos et al. (2022), He et al. (2021), and Yildiz et al. (2019) showed a complete enthesis healing with hDCB-TDSCs-ECM and HDG/TFL. Moreover, He et al. (2021) performed micro-CT for BV/TV and trabecular thickness (Tb.Th) measurements at the tendon-to-bone insertion, evidencing an increase in these parameters in the hDCB-ECM group compared to untreated controls.

Biomechanical tests were conducted by most of the research groups (67%): all experimental groups exhibited higher tensile stress (He et al., 2021) and strength values (Yildiz et al., 2019; He et al., 2021; Yuan et al., 2022b; Yuan et al., 2022a), failure loads (Yuan et al., 2022a; Yuan et al., 2022b), and cyclical loading values (Yildiz et al., 2019) with increasing experimental times in comparison to control groups, except for one study in which decellularized patches failed to load, evidencing lower biomechanical competence than the untreated control group (Yildiz et al., 2019).

#### 3.2.3 Dogs

Two studies out of 13 (15%) used canine models: Smith et al. (2020) performed half-thickness resection of the articular portion of SST (3.7 x 3–4 mm) in 2–3-year-old purpose-bred dogs, whereas Chen et al. (2022) executed a unilateral IST detachment from the insertion at the humerus in 8-month-old beagle dogs. The SST defect was left untreated in the control group or treated with different commercial matrices: amnion matrix cord scaffold (AM from Arthrex, Inc.), decellularized human dermal allograft (ArthroFLEX (AF) from Arthrex, Inc./LifeNet Health, Virginia Beach, VA, United States), and bovine collagen patch (rotation medical patch, RMP, from Smith & Nephew, London, United Kingdom) (Smith et al., 2020). Chen et al. (2022) developed a collagen-binding peptide (CBP)-growth factor (GF)-decellularized enthesis matrix (O-BDEM) loaded with or without urine-derived stem cells (USCs) (Smith et al., 2020). Only one reported the sex of animals, with experimental times ranging between 3 and 6 months (Smith et al., 2020; Chen et al., 2022).

Both research groups performed MRI and biomechanical testing to, respectively, assess the repaired area (Smith et al., 2020) and tendon and muscle conditions (Smith et al., 2020) by measuring tendon tensile loading (Smith et al., 2020), failure load (Chen et al., 2022), and stiffness (Smith et al., 2020; Chen et al., 2022). MRI in Smith et al. (2020) evidenced the bridging of the SST defect in AM, AF, and RMP groups, but hyper-intense peritendon, intramuscular, intra-articular fluid, and impingement of biceps were found in all groups at 3 months, and impingement of biceps was increased at 6 months, especially in the AF group. In the work of Chen et al. (2022), a better regeneration of IST was observed in the experimental group compared to the control group. In addition, Smith et al. (2020) and Chen et al. (2022) performed ultrasonography and micro-CT investigations, respectively: AM-treated and AF-treated SSTs were thinner but showed more organized tendon fiber alignment than the other groups at 6 months (Johnson et al., 2020); bone parameters BV/TV, Tb.Th, and Tb.N have improved in the treated group after patch augmentation in comparison with untreated controls (Chen et al., 2022).

Biomechanical parameters were improved in the experimental group compared to controls in Chen et al.’s (2022) study, while no significant differences were observed between experimental groups in the work of Smith et al. (2020). Moreover, macroscopic analyses were performed to examine the gap healing rate and SST integrity: in one study, no significant differences were observed between groups (Chen et al., 2022), whereas in the other study, SSTs were partially to fully intact in all groups at each time point (Smith et al., 2020).

Smith et al. (2020) analyzed the lameness grade and the level of forelimb function at trot, assessed arthroscopically the articular cartilage, biceps tendon impingement, synovium, SSTs, and lateral glenohumeral ligament, and used comfortable shoulder range of motion (CROM) and visual analog scale (VAS) pain scores to compare groups: all treatment groups exhibited significantly lower CROM and higher VAS pain scores at each time point, especially DB and RMP groups compared to controls, and a most severe synovitis, fibrosis, and biceps tendon impingement in the DB group at 3 months and least severe in AF and AM groups at 6 months after surgery.

#### 3.2.4 Sheep

Rambouillet cross sheep, 2–3 years old, were adopted as a preclinical model of chronic rotator cuff (RC) degeneration by Credille et al. This group realized a chronic model of full-thickness IST degeneration in the right shoulder and reconstructed the tear after 6 weeks of surgery by augmentation with a biphasic interpositional decellularized trabecular bone allograft.

A qualitative macroscopic examination was conducted on the front limbs, all major organs, scar tissue, tendon retraction, and muscle atrophy. Gross pathology evidenced a thickening of ISTs and their covering with fibrotic scar tissue after 6 weeks of tendon degeneration (Credille et al., 2023).

### 3.3 Risk of bias assessment

**FIGURE 2:**
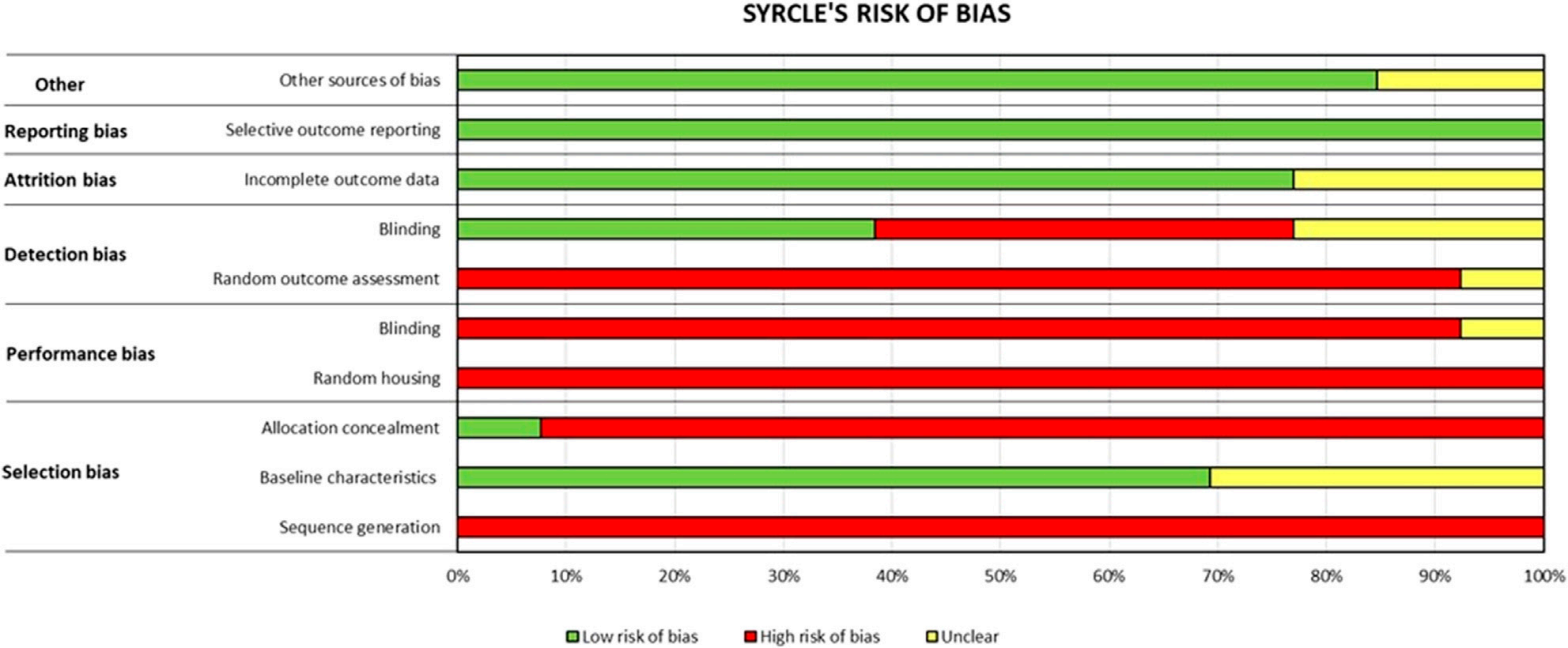
Risk of bias assessment of each *in vivo* paper by applying the SYRCLE tool (de Andrade et al., 2022). The frequency % of each item is reported as high risk of bias (red bar), low risk of bias (green bar), and unclear risk of bias (yellow bar).

The risk of bias was assessed using the SYRCLE tool, and it is shown in Figure 2. The risk of bias resulted high for most items, such as items 1 (sequence generation), 3 (allocation concealment), 4 (random housing), 5 (blinding), and 6 (random outcome assessment), with respective frequencies of 100%, 92%, 100%, 92%, and 92%. There was a low risk of bias for items 2 (baseline characteristics), 8 (incomplete outcome data), 9 (selective outcome reporting), and 10 (other sources of bias) with frequencies of 69%, 77%, 100%, and 85%, respectively. Item 7 (blinding) resulted in an unclear risk of bias. Raw data are reported in Supplementary Table S1.

## 4 Discussion

The aim of this systematic review is to provide an overview of the literature on the efficacy of biological patches applied using the augmentation technique for RCT treatment. Decellularized patches for RC augmentation are biological (human or animal) acellular matrices (autograft, allograft, and xenograft) used for their ability to induce native tissue growth, promote RC healing, and provide biomechanical support (Zumstein et al., 2017; Cobb et al., 2022).

Our review identifies a small number of papers investigating the efficacy of decellularized patches in animal models of RCTs. This important aspect is related to the anatomical and biomechanical differences that exist between the human and animal scapulohumeral joints: in humans, the scapulohumeral joint is characterized by the most varied and extensive movements compared to other quadrupedal mammals, in which the arm does not detach from the trunk or detaches very little and in which abduction, adduction, circumduction, and rotation movements are very limited. There are relatively few *in vivo* studies of RC repair in the bibliography. This is because only non-human primates, albeit with ethical and legal limitations, have the same biomechanical characteristics as humans. Therefore, the *in vivo* RC repair model is not truly translational to what happens in humans. As evidenced, spontaneous shoulder injuries are very rare in veterinary medicine.

Another concern is related to the evaluation of efficacy; in fact, unlike clinical studies, where MRI and ultrasound imaging investigations are routinely used to assess the healing or re-tear rate, in *in vivo* studies, there is no gold standard for the outcome. Only three studies (two in dogs and one in rats) performed MRI and/or ultrasound evaluations, while in the remaining studies, different macroscopic, histological, and histomorphometric scoring systems and measurements were applied. For this reason, comparing studies is difficult. Despite the requirement for sample harvest, these analyses allow for the determination of a variety of aspects related to the biological response of tissues to decellularized patches (Maglio et al., 2020), such as the formation of new fibrocartilage tissue at the insertion site, enthesis maturation, implant degradation, organization, and orientation of newly deposited collagen fibers, as well as the presence of inflammatory infiltrates or the formation of new blood vessels. In addition, the evaluation of the expression levels of genes such as *COL1*, *COL2*, *COL3*, *ACAN*, and *TNC* after RNA extraction from tissues may provide additional useful information on RC healing before and after treatment (Yuan et al., 2022a; Yuan et al., 2022b; Pagani et al., 2023). No gait analysis is performed, except for a paper assessing the degree of lameness and functionality of the forelimbs (Smith et al., 2020); this test is performed for locomotion analysis and allows longitudinal repeated measurements, sequentially or at different time points, without the need to restrain the animal, thus ethically complying with the principles of animal reduction and refinement. Pain scales are widely used in clinical practice to quantify pain severity, such as the VAS for the subjective measurement of pain experienced by patients.

Only Smith et al. (2020) assessed pain severity using validated CROM and VAS scales in dogs although many human acute pain scales, such as the composite scale (CS), numerical rating scale (NRS), and simple descriptive scale (SDS), are adopted for animal species to establish appropriate pain therapy by ensuring animal welfare (Bufalari et al., 2007; Hielm-Björkman, 2013; Bianchi et al., 2023). Moreover, the Grimace scale is used to evaluate pain in different animal models (https://www.nc3rs.org.uk/3rs-resources/grimace-scales) (Häger et al., 2017); the use of these scales is desired and has to be improved even to accomplish one of the 3R principles in terms of refinement (Russell and Burch, 1959). The review shows that the rabbit is the most used animal model for this type of study, followed by the rat. The former is mainly used to understand the mechanisms underlying the muscle changes associated with RCTs, while the latter is used to better understand the potential mechanisms of RC injury due to the anatomical analogy with the SST tendon (Edelstein et al., 2011). Small animal models have many advantages, including ease of management, but they also have limitations in terms of clinical translation and surgical procedures. Larger animal models, such as sheep and dogs, have been used to overcome these problems, albeit to a lesser extent. Smith et al. and Chen et al. used a canine model for their studies due to the reliability of performance and biological regenerative patterns (Yang et al., 2022). However, the use of canine models is limited in Europe due to ethical and legal concerns (Martini et al., 2001). Instead, Credille et al. used sheep because the ovine shoulder girdle is similar to the human IST and SST and is a commonly used model for orthopedic research (Martini et al., 2001).

None of the included studies analyzed sex differences because they did not test the efficacy of the patches in both sexes simultaneously; the research groups either used only male animals (Ide and Tokunaga, 2018; Chen et al., 2020; Chen et al., 2022; de Lima Santos et al., 2020; He et al., 2021; Santos et al., 2022) or, to a lesser extent, female animals (Thangarajah et al., 2018; Credille et al., 2023). It is now well established that sex differences, mainly due to hormonal variations and levels, affect musculoskeletal pathologies, including tendons (Contartese et al., 2020; Tschon et al., 2021; Mondini Trissino Da Lodi et al., 2022; Salamanna et al., 2022; Salamanna et al., 2023). Clinical studies involving both men and women have shown a difference in prevalence (Razmjou et al., 2016; Sabo et al., 2021) on the extent of shoulder pathology, repair, and healing, with a higher rate of re-tear in women than in men (Collin et al., 2015; Razmjou et al., 2016).

Regarding the surgically induced injury, two types of surgery have been performed: unilateral or bilateral tendon detachment (Thangarajah et al., 2018; Chen et al., 2020; Chen et al., 2022; He et al., 2021) and the creation of tendon–bone defects (Ide and Tokunaga, 2018; Yildiz et al., 2019; de Lima Santos et al., 2020; Smith et al., 2020; Yuan et al., 2022a; Yuan et al., 2022b; Santos et al., 2022; Shim et al., 2022). In rats and rabbits, the average size of the induced tear is 5 mm in length (Ide and Tokunaga, 2018; Yuan et al., 2022a; Yuan et al., 2022b; Santos et al., 2022), whereas in dogs it is 7.5 ± 1.0 mm (Smith et al., 2020). In rabbits, several studies find that RCTs > 5 mm in diameter could not heal spontaneously, whereas no specific determinations of lesion size as critical have been reported for larger animal models (Sabo et al., 2021).

As reported in the literature, these sizes of RCTs are compatible with partial or full-thickness moderate and large-to-massive rotator cuff injuries seen in humans (Liu et al., 2011; Onay, 2013; Ditsios et al., 2014; Redler et al., 2014; Kataoka et al., 2018; Kwon et al., 2018; Zhao et al., 2022).

In humans, pathologies affecting RC are usually chronic lesions associated with myotendinous retraction, atrophy, and fatty infiltration of the muscles. In *in vivo* preclinical testing, there are difficulties in reproducing these degenerative alterations (Sevivas et al., 2015). Several animal models have been widely used to emphasize the etiology or pathogenesis of RC disorders and assess innovative experimental approaches. Tendon detachment in the rat leads to degenerative changes such as tendon degeneration, inflammation, and muscle atrophy comparable to those seen in the clinical setting. Moreover, in this model, Thangarajah et al. (2017) found a reduction in BMD at the enthesis that could be helpful for innovative bone fixation device evaluation. On the other hand, in a study of rat SST detachment, Barton et al. (2005) demonstrated the spontaneous healing of the tendon. In addition, unilateral or bilateral detachment has also been used as a surgical technique to create RCTs although it has been shown that the amount of scar tissue formed during the injury repair process leads to permanent gait impairment and interferes with the healing process itself (Theodossiou and Schiele, 2019). So far, the defect creation could overcome some of the limitations of the tendon detachment, leading to tears of different sizes, mimicking large-to-massive RC defects, as in the clinical setting. In addition, animal models of RC defects allow for the implantation of experimental scaffolds, such as in cases of decellularized soft membranes. Almost all authors realized an acute model of RC injury, as tendon injuries/defects are treated immediately in the same surgical session.

Thangarajah et al. (2018), Yildiz et al. (2019), Shim et al. (2022), and Credille et al. (2023) realized a chronic model of RCTs, which is a condition more like human tendinopathy. It consists of a two-step surgery to induce the RC defects and treat them within a timeframe ranging from 3 weeks to 8 weeks after the first surgery. The article by Sengupta (2013) correlated the age of rats and rabbits to that of humans and claimed that 1 month of life of a rat and rabbit equals 3 and 1 human year, respectively, since they undergo very rapid growth in the early life stages (Sengupta and Dutta, 2020). According to Abdou et al. (2019), 4 weeks from the first to the second surgery is the suitable timeframe to make a chronic RCT rabbit model. It should also be considered that, in contrast to the arthroscopic minimally invasive approach in the human clinical setting, all animal surgeries are performed in an open setting (Modi et al., 2022).

**FIGURE 3:**
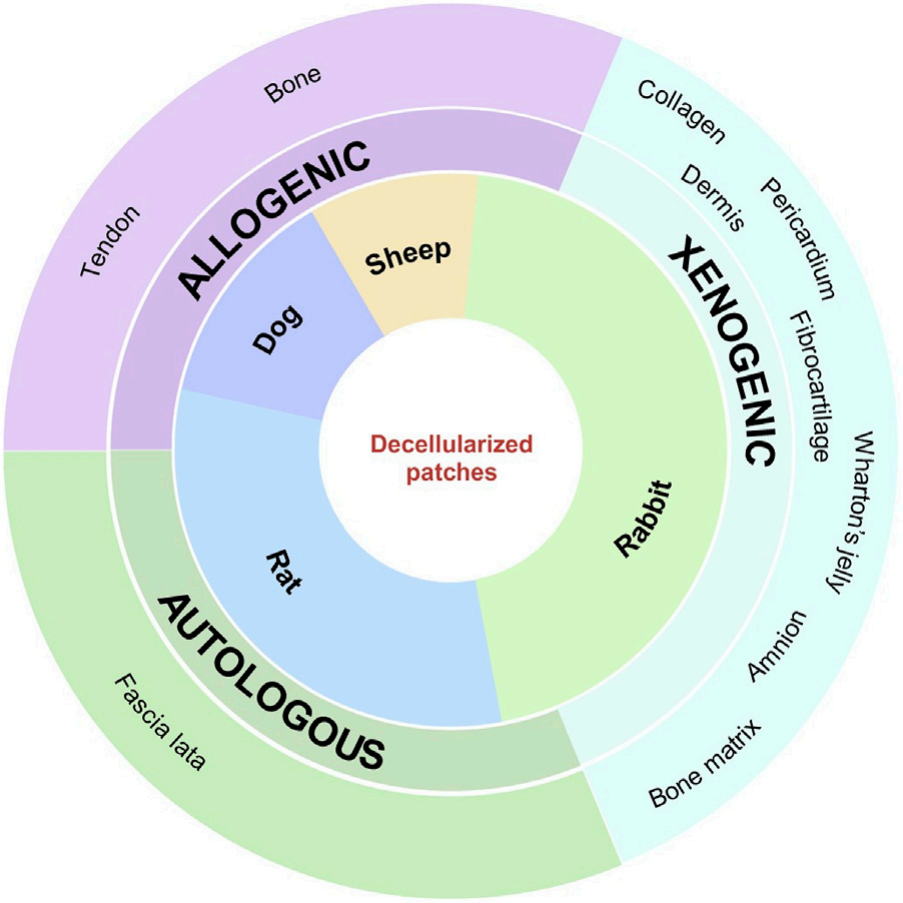
Image depicting the animal species used in the included articles, with the indication of the main types and sources of different acellular patches.

Allogeneic and xenogeneic matrices are the most commonly used decellularized patches: half of the rat studies use a commercially available human dermal matrix, GraftJacket™ (Ide and Tokunaga, 2018; Thangarajah et al., 2018), which provides support and protection for ligaments and tendons, demonstrates improvements in pain, range of motion, and strength, and has been shown in various clinical trials to be effective in the treatment of irreparable RCTs with a low risk of re-tear (Wong et al., 2010; Barber et al., 2012; Sharma et al., 2018; Johnson et al., 2020; Modi et al., 2022). The decellularization protocol for this patch is not reported, as it is a registered trademark. Smith et al. (2020) compared the efficacy of three different commercial matrices for RC tendon defects in their study: amniotic membrane scaffold (AM), decellularized human dermal allograft (AF), and bovine collagen patch (RMP). AF shows the best results in terms of collagen fiber organization, patch integration at the insertion site, fibrosis, synovitis, biceps tendon impingement, and less severe pathology compared to the other matrices tested. As with GraftJacket™, the use of a commercial product ensures lower variability, greater reproducibility, and fewer adverse effects and may have a higher chance of success for RCT repair. In addition, AF is now being investigated in two clinical trials registered at https://clinicaltrials.gov/website. The other studies used different decellularized matrices derived from the bovine pericardium (Shim et al., 2022), rabbit pubic symphysis (Chen et al., 2020), rabbit gastrocnemius (de Lima Santos et al., 2020; Santos et al., 2022), human cortical or trabecular bone (He et al., 2021; Credille et al., 2023), human tensor fascia lata (Yildiz et al., 2019), human umbilical cord (Yuan et al., 2022b; Yuan et al., 2022a), and canine IST (Chen et al., 2022), as represented in Figure 3. Their main limitation is the high variability due to both a non-standardized decellularization protocol and the different animal models used, which do not help predict their success or failure rates in a clinical trial. None of these non-commercial patches are currently being investigated for the treatment of RCTs in clinical trials.

Regarding the quality of the included papers, the main limitations are related to the experimental design, as most papers do not report randomization of allocation and outcome measures, blinding, and allocation concealment, which increases the risk of bias in their studies. For example, less than half of the research teams performed an *a priori* analysis to determine the sample size (Thangarajah et al., 2018; Yildiz et al., 2019; de Lima Santos et al., 2020; Chen et al., 2022; Santos et al., 2022; Shim et al., 2022), although the use of a large sample size without an *a priori* analysis (Yuan et al., 2022a; Yuan et al., 2022b; Credille et al., 2023) could not ensure compliance with the ethical requirement of reduction (Russell and Burch, 1959).

This systematic review presents several limitations. First, we limit our search to the last 5 years although the search is performed strictly in accordance with the PRISMA guidelines, by registering our protocol in the public register PROSPERO at the beginning and by using an international tool to assess the risk of bias. The second drawback is related to the efficacy outcome measurement: our review of preclinical models shows that no gold standard technique is used, thus making studies inhomogenous and hampering the absolute recognition of the most suitable and effective decellularized patch. Inherently to the animal models, open surgery is mainly performed compared to arthroscopic procedures performed in the clinical scenario, and the animal posture is different than the human counterpart because in quadrupeds tendons are subjected to different forces and loads (Hast et al., 2014). When conducting a study aimed at investigating surgical and orthopedic problems, it is essential to carefully select an animal model that is as similar as possible to the human in terms of anatomy, affected area, and size. In this context, it is important to emphasize that no animal model, with the exception of non-human primates, can fully replicate the repair mechanisms and physiological conditions associated with human RC injury, healing, and regeneration due to the quadrupedal posture (Yang et al., 2022). Long-term follow-up is lacking for most of the reported augmentation options, and future studies with mid- and long-term follow-up are warranted.

Despite these limitations, this review provides a comprehensive overview of the use of acellular patches and their therapeutic potential in RC repair at the preclinical level, with the aim of expanding the strategies and matrices available to surgeons.

## Data Availability

The original contributions presented in the study are included in the article/Supplementary Material; further inquiries can be directed to the corresponding author.
