# Supplementary material for: Decellularized biological matrices for the repair of rotator cuff lesions: a systematic review of preclinical in vivo studies
Source: Front Bioeng Biotechnol. 2024 Feb 1;12:1345343. doi: 10.3389/fbioe.2024.1345343 (PMC10867272; doi:10.3389/fbioe.2024.1345343)
Supplement: Supplementary file 1 [file Table1.docx]

Supplementary Material

## Table 1. List of excluded papers with reasons.

| **Article** | **Reason for exclusion** |
| --- | --- |
| Tovar-Bazaga M, Pérez-Cuesta Llaneras M, Badia A. Reconstruction of delayed distal biceps ruptures with a dermal matrix. Hand Surg Rehabil. 2023 Jun;42(3):243-249. doi: 10.1016/j.hansur.2023.03.004. Epub 2023 Mar 31. PMID: 37004984. | Clinical study |
| Castells-Sala C, Pérez ML, López-Chicón P, Lopez-Puerto L, Martinez JIR, Ruiz-Ponsell L, Aiti A, Madariaga SE, Sastre S, Fariñas O, Vilarrodona A. Development of a full-thickness acellular dermal graft from human skin: Case report of first patient rotator cuff patch augmentation repair. Transpl Immunol. 2023 Jun;78:101825. doi: 10.1016/j.trim.2023.101825. Epub 2023 Mar 18. PMID: 36934900. | No preclinical models |
| Lee S, Koh KH, Shin SJ. Superior Capsule Reconstruction Using an Acellular Dermal Matrix Allograft Combined With Remnant Tendon Augmentation for Irreparable Rotator Cuff Tear. Arthrosc Tech. 2023 Jan 31;12(2):e241-e246. doi: 10.1016/j.eats.2022.10.013. PMID: 36879872; PMCID: PMC9984778. | Technical note |
| Mihata T. Editorial Commentary: Superior Capsule Reconstruction: Acellular Allograft at 45° of Glenohumeral Abduction Improves Glenohumeral Stability, but Fascia Lata Autograft Remains Superior. Arthroscopy. 2023 Apr;39(4):931-934. doi: 10.1016/j.arthro.2022.12.020. PMID: 36872033. | Editorial commentary |
| Jackson GR, Tuthill T, Schundler SF, Condon JJ, Salazar LM, Nwiloh M, Kaplan DJ, Brusalis CM, Khan ZA, Knapik DM, Chahla J, Cole BJ, Verma NN. Acellular Dermal Allograft and Tensor Fascia Lata Autograft Show Similar Patient Outcome Improvement and High Rates of Complications and Failures at a Minimum 2-Year Follow-Up: A Systematic Review. Arthroscopy. 2023 May;39(5):1310-1319.e2. doi: 10.1016/j.arthro.2023.01.003. Epub 2023 Jan 16. PMID: 36657648. | Review |
| Suri M, Parry S, Dham M, Verma A. Arthroscopic Biologic Tuberoplasty for Irreparable Rotator Cuff Tears: An Expedited Technique. Arthrosc Tech. 2022 Nov 17;11(12):e2265-e2270. doi: 10.1016/j.eats.2022.08.035. PMID: 36632403; PMCID: PMC9827058. | Technical note |
| Perry NPJ, Farina EM, Wang C, Price MD, Mazzocca AD. Editorial Commentary: Fascia Lata Allograft for Shoulder Superior Capsular Reconstruction: In the Fight Over Optimal Graft Choice for Irreparable Rotator Cuff Tears, Superior Capsular Reconstruction Proponents May Be Changing Their Gloves. Arthroscopy. 2023 Jan;39(1):29-31. doi: 10.1016/j.arthro.2022.09.019. PMID: 36543419. | Editorial commentary |
| Morgan CN, Bonner KF, Griffin JW. Augmentation of Arthroscopic Rotator Cuff Repair: Biologics and Grafts. Clin Sports Med. 2023 Jan;42(1):95-107. doi: 10.1016/j.csm.2022.08.010. PMID: 36375873. | Review |
| Altintas B, Storaci HW, Lacheta L, Dornan GJ, Krob JJ, Aman ZS, Anderson N, Rosenberg SI, Millett PJ. Superior Capsule Reconstruction Using Acellular Dermal Allograft Secured at 45° of Glenohumeral Abduction Improves the Superior Stability of the Glenohumeral Joint in Irreparable Massive Posterosuperior Rotator Cuff Tears. Arthroscopy. 2023 Apr;39(4):922-930. doi: 10.1016/j.arthro.2022.10.037. Epub 2022 Nov 4. PMID: 36343768. | Cadaveric study (No preclinical models) |
| Bai L, Han Q, Meng Z, Chen B, Qu X, Xu M, Su Y, Qiu Z, Xue Y, He J, Zhang J, Yin Z. Bioprinted living tissue constructs with layer-specific, growth factor-loaded microspheres for improved enthesis healing of a rotator cuff. Acta Biomater. 2022 Dec;154:275-289. doi: 10.1016/j.actbio.2022.10.058. Epub 2022 Nov 1. Erratum in: Acta Biomater. 2023 Mar 15;159:423. PMID: 36328126. | No decellularized patches |
| Ankem HK. Editorial Commentary: Allogenic Dermal Fibroblasts in Collagen Matrix Scaffold Enhance Rotator Cuff Repair in an Animal Model. Arthroscopy. 2022 Jul;38(7):2129-2130. doi: 10.1016/j.arthro.2022.01.030. PMID: 35809975. | Editorial commentary |
| O'Neil S, Marvil S, Lakehomer H, Tipton CC, Dobrich J, Gilmer BB, Guttmann D. Modified Technique for Arthroscopic Bursal Acromial Reconstruction Utilizing Acellular Dermal Allograft. Arthrosc Tech. 2022 Feb 13;11(3):e301-e306. doi: 10.1016/j.eats.2021.10.022. PMID: 35256967; PMCID: PMC8897560. | Technical note |
| Tauro TM, Wagner KR, DeFroda SF, Muth S, Bodendorfer BM, Verma NN, Cole BJ. Technical Note: Arthroscopic Rotator Cuff Repair with Patch Augmentation with Acellular Dermal Allograft. Arthrosc Tech. 2022 Jan 6;11(2):e121-e125. doi: 10.1016/j.eats.2021.09.011. PMID: 35155102; PMCID: PMC8820992. | Technical note |
| Scardina L, DI Leone A, Sanchez AM, D'Archi S, Biondi E, Franco A, Mason EJ, Magno S, Terribile D, Barone-Adesi L, Visconti G, Salgarello M, Masetti R, Franceschini G. Nipple sparing mastectomy with prepectoral immediate prosthetic reconstruction without acellular dermal matrices: a single center experience. Minerva Surg. 2021 Dec;76(6):498-505. doi: 10.23736/S2724-5691.21.08998-X. PMID: 34935320. | No rotator cuff |
| Forlizzi JM, Sylvia SM, Pettit RJ, Saini SS, MacAskill M, Ross G, Shah SS. Surgical Technique for Superior Capsule Reconstruction With 6-mm Acellular Dermal Allograft and Knotless Glenoid Anchors. Arthrosc Tech. 2021 Jun 19;10(7):e1821-e1827. doi: 10.1016/j.eats.2021.04.001. PMID: 34336581; PMCID: PMC8322625. | Technical note |
| Berthold DP, Ravenscroft M, Bell R, Obopilwe E, Cote MP, Kane Z, Morgan BW, Mühlenfeld N, Mazzocca AD, Muench LN. Bursal Acromial Reconstruction (BAR) Using an Acellular Dermal Allograft for Massive, Irreparable Posterosuperior Rotator Cuff Tears: A Dynamic Biomechanical Investigation. Arthroscopy. 2022 Feb;38(2):297-306.e2. doi: 10.1016/j.arthro.2021.07.021. Epub 2021 Jul 28. PMID: 34329702. | Cadaveric study (no preclinical models) |
| Tomarchio A, Meccariello L, Ghargozloo D, Pasquino A, Leonardi E. Relapses of traumatic peroneal tendons subluxation already treated surgically: a new surgical approach. Med Glas (Zenica). 2021 Aug 1;18(2):487-492. doi: 10.17392/1354-21. PMID: 34308618. | Clinical study |
| Condron NB, Kester BS, Tokish JM, Zumstein MA, Gobezie R, Scheibel M, Cole BJ. Nonoperative and Operative Soft-Tissue, Cartilage, and Bony Regeneration and Orthopaedic Biologics of the Shoulder: An Orthoregeneration Network (ON) Foundation Review. Arthroscopy. 2021 Oct;37(10):3200-3218. doi: 10.1016/j.arthro.2021.06.033. Epub 2021 Jul 20. PMID: 34293441. | Review |
| Ezagüi Bentolila L. Surgical Technique for Arthroscopic Rotator Cuff Augmentation With Human Acellular Dermal Matrix. Arthrosc Tech. 2021 Mar 8;10(4):e1025-e1032. doi: 10.1016/j.eats.2020.12.002. PMID: 33981546; PMCID: PMC8085310. | Technical note |
| Haque A, Modi A. Interposition grafting for irreparable rotator cuff tears: Systematic review and specialist practice report. J Clin Orthop Trauma. 2021 Mar 13;17:218-222. doi: 10.1016/j.jcot.2021.02.022. PMID: 33868918; PMCID: PMC8047223. | Review |
| Lee SJ, Rabinovich RV, Kim A. Proximal Row Carpectomy Using Decellularized Dermal Allograft: Preliminary Results. J Wrist Surg. 2021 Apr;10(2):116-122. doi: 10.1055/s-0040-1718912. Epub 2020 Oct 29. PMID: 33815946; PMCID: PMC8012091. | No rotator cuff |
| Ravenscroft M, Barnes MW, Muench LN, Mazzocca AD, Berthold DP. Bursal Acromial Reconstruction (BAR) Using an Acellular Dermal Allograft as a Surgical Solution for the Treatment of Massive Irreparable Rotator Cuff Tears. Arthrosc Tech. 2021 Feb 22;10(3):e877-e885. doi: 10.1016/j.eats.2020.11.002. PMID: 33738228; PMCID: PMC7953327. | Technical note |
| Lee JS, Park E, Lee JH, Lee J, Park HY, Yang JD, Jung TD. A prospective comparison study of early functional outcomes after implant-based breast reconstruction: subpectoral versus prepectoral technique. Ann Palliat Med. 2021 Mar;10(3):2520-2529. doi: 10.21037/apm-20-1550. Epub 2021 Feb 26. PMID: 33691448. | No rotator cuff |
| Mirzayan R, Otarodifard KA, Singh A. Arthroscopic Superior Capsule Reconstruction with a Doubled-Over (6 mm) Dermal Allograft. Arthrosc Tech. 2021 Jan 21;10(2):e525-e530. doi: 10.1016/j.eats.2020.10.035. PMID: 33680787; PMCID: PMC7917301. | Technical note |
| Franceschini G, Scardina L, Di Leone A, Terribile DA, Sanchez AM, Magno S, D'Archi S, Franco A, Mason EJ, Carnassale B, Murando F, Orlandi A, Barone Adesi L, Visconti G, Salgarello M, Masetti R. Immediate Prosthetic Breast Reconstruction after Nipple-Sparing Mastectomy: Traditional Subpectoral Technique versus Direct-to-Implant Prepectoral Reconstruction without Acellular Dermal Matrix. J Pers Med. 2021 Feb 22;11(2):153. doi: 10.3390/jpm11020153. PMID: 33671712; PMCID: PMC7926428. | No rotator cuff |
| Shi Q, Chen C, Li M, Chen Y, Xu Y, Hu J, Liu J, Lu H. Characterization of the distributions of collagen and PGs content in the decellularized book-shaped enthesis scaffolds by SR-FTIR. BMC Musculoskelet Disord. 2021 Mar 1;22(1):235. doi: 10.1186/s12891-021-04106-x. PMID: 33648475; PMCID: PMC7923620. | No preclinical model |
| Smith TJ, Gowd AK, Kunkel J, Kaplin L, Hubbard JB, Coates KE, Graves BR, Waterman BR. Clinical Outcomes of Superior Capsular Reconstruction for Massive, Irreparable Rotator Cuff Tears: A Systematic Review Comparing Acellular Dermal Allograft and Autograft Fascia Lata. Arthrosc Sports Med Rehabil. 2020 Dec 27;3(1):e257-e268. doi: 10.1016/j.asmr.2020.09.002. PMID: 33615273; PMCID: PMC7879184. | Review |
| Chen W, Sun Y, Gu X, Cai J, Liu X, Zhang X, Chen J, Hao Y, Chen S. Conditioned medium of human bone marrow-derived stem cells promotes tendon-bone healing of the rotator cuff in a rat model. Biomaterials. 2021 Apr;271:120714. doi: 10.1016/j.biomaterials.2021.120714. Epub 2021 Feb 11. PMID: 33610048. | No decellularized patches |
| Wattoo G, Nayak S, Khan S, Morgan J, Hocking H, MacInnes E, Kolar KM, Rogers C, Olubowale O, Rigby K, Kazzazi NH, Wyld L. Long-term outcomes of latissimus dorsi flap breast reconstructions: A single-centre observational cohort study with up to 12 years of follow up. J Plast Reconstr Aesthet Surg. 2021 Sep;74(9):2202-2209. doi: 10.1016/j.bjps.2020.12.058. Epub 2020 Dec 26. PMID: 33451948. | No rotator cuff |
| Shah SS, Kontaxis A, Jahandar A, Bachner E, Gulotta LV, Dines DM, Warren RF, Dines JS, Taylor SA. Superior capsule reconstruction using a single 6-mm-thick acellular dermal allograft for massive rotator cuff tears: a biomechanical cadaveric comparison to fascia lata allograft. J Shoulder Elbow Surg. 2021 Sep;30(9):2166-2176. doi: 10.1016/j.jse.2020.11.015. Epub 2021 Jan 5. PMID: 33418091. | *In vitro* study |
| Shi Q, Chen Y, Li M, Zhang T, Ding S, Xu Y, Hu J, Chen C, Lu H. Designing a novel vacuum aspiration system to decellularize large-size enthesis with preservation of physicochemical and biological properties. Ann Transl Med. 2020 Nov;8(21):1364. doi: 10.21037/atm-20-3661. PMID: 33313109; PMCID: PMC7723548. | No rotator cuff |
| Hirahara AM, Andersen WJ, Dooney T. Arthroscopic Knotless Rotator Cuff Repair With Decellularized Dermal Allograft Augmentation: The "Canopy" Technique. Arthrosc Tech. 2020 Oct 21;9(11):e1797-e1803. doi: 10.1016/j.eats.2020.08.003. PMID: 33294343; PMCID: PMC7695626. | Technical note |
| Chae S, Sun Y, Choi YJ, Ha DH, Jeon I, Cho DW. 3D cell-printing of tendon-bone interface using tissue-derived extracellular matrix bioinks for chronic rotator cuff repair. Biofabrication. 2021 Apr 2;13(3). doi: 10.1088/1758-5090/abd159. PMID: 33285539. | No decellularized patches |
| Gao I, Sochacki KR, Freehill MT, Sherman SL, Abrams GD. Superior Capsular Reconstruction: A Systematic Review of Surgical Techniques and Clinical Outcomes. Arthroscopy. 2021 Feb;37(2):720-746. doi: 10.1016/j.arthro.2020.09.016. Epub 2020 Nov 20. PMID: 33227320. | Review |
| Dyrna F, Berthold DP, Muench LN, Beitzel K, Kia C, Obopilwe E, Pauzenberger L, Adams CR, Cote MP, Scheiderer B, Mazzocca AD. Graft Tensioning in Superior Capsular Reconstruction Improves Glenohumeral Joint Kinematics in Massive Irreparable Rotator Cuff Tears: A Biomechanical Study of the Influence of Superior Capsular Reconstruction on Dynamic Shoulder Abduction. Orthop J Sports Med. 2020 Oct 6;8(10):2325967120957424. doi: 10.1177/2325967120957424. PMID: 33088839; PMCID: PMC7543188. | Cadaveric study (no preclinical models) |
| Wang Z, Long Z, Amadio PC, Gingery A, Moran SL, Steinmann SP, Zhao C. Biomechanical Comparison of Augmentation of Engineered Tendon-Fibrocartilage-Bone Composite With Acellular Dermal Graft Using Double Rip-Stop Technique for Canine Rotator Cuff Repair. Orthop J Sports Med. 2020 Sep 2;8(9):2325967120939001. doi: 10.1177/2325967120939001. PMID: 32953920; PMCID: PMC7476351. | No preclinical models |
| Chalmers PN, Tashjian RZ. Patch Augmentation in Rotator Cuff Repair. Curr Rev Musculoskelet Med. 2020 Oct;13(5):561-571. doi: 10.1007/s12178-020-09658-4. PMID: 32720101; PMCID: PMC7474721. | Review |
| Prokuski V, Strohl A. Soft Tissue Coverage for Severe Infections. Hand Clin. 2020 Aug;36(3):369-379. doi: 10.1016/j.hcl.2020.03.011. PMID: 32586464. | Review |
| Stone AV, Luo TD, Sharma A, Danelson KA, De Gregorio M, Freehill MT. Optimizing the Double-Row Construct: An Untied Medial Row Demonstrates Equivalent Mean Contact Pressures in a Rotator Cuff Model. Orthop J Sports Med. 2020 Apr 27;8(4):2325967120914932. doi: 10.1177/2325967120914932. PMID: 32426405; PMCID: PMC7218996. | No preclinical models |
| Dyrberg DL, Gunnarsson GL, Bille C, Sørensen JA, Thomsen JB. Direct-to-Implant Extracellular Matrix Hammock-based Breast Reconstruction; Prepectoral or Subpectoral? Trials. 2020 Feb 10;21(1):160. doi: 10.1186/s13063-020-4125-6. PMID: 32041661; PMCID: PMC7011213. | No rotator cuff |
| Matthewson G, Coady CM, Wong IH. Rotator Cuff Reconstruction Using Fascia Lata Patch Autograft for the Nonrepairable Rotator Cuff Tear. Arthrosc Tech. 2020 Jan 23;9(1):e123-e130. doi: 10.1016/j.eats.2019.09.006. PMID: 32021785; PMCID: PMC6993264. | Technical note |
| Lee TQ. Editorial Commentary: Biomechanical Investigation of Superior Capsule Reconstruction Requires Meticulous Methods. Arthroscopy. 2020 Feb;36(2):365-366. doi: 10.1016/j.arthro.2019.10.037. PMID: 32014170. | Editorial commentary |
| Meaike JJ, Patterson DC, Anthony SG, Parsons BO, Cagle PJ. Soft tissue resurfacing for glenohumeral arthritis: a systematic review. Shoulder Elbow. 2020 Feb;12(1):3-11. doi: 10.1177/1758573219849606. Epub 2019 May 31. PMID: 32010227; PMCID: PMC6974882. | Review |
| John R, Coady CM, Wong I. Revision of a Failed Latissimus Dorsi Transfer for a Massive Rotator Cuff Tear With Arthroscopic Anatomic Bridging Reconstruction Using an Acellular Human Dermal Matrix Allograft. Arthrosc Tech. 2019 Sep 19;8(10):e1171-e1179. doi: 10.1016/j.eats.2019.06.007. PMID: 31921592; PMCID: PMC6950775. | Technical note |
| Adams CR, Comer B, Scheiderer B, Imhoff FB, Morikawa D, Kia C, Muench LN, Baldino JB, Mazzocca AD. The Effect of Glenohumeral Fixation Angle on Deltoid Function During Superior Capsule Reconstruction: A Biomechanical Investigation. Arthroscopy. 2020 Feb;36(2):400-408. doi: 10.1016/j.arthro.2019.09.011. Epub 2020 Jan 2. PMID: 31902546. | Cadaveric study (no preclinical models) |
| Pascual-Garrido C, Schwabe MT, Chahla J, Haneda M. Surgical Treatment of Gluteus Medius Tears Augmented With Allograft Human Dermis. Arthrosc Tech. 2019 Oct 25;8(11):e1379-e1387. doi: 10.1016/j.eats.2019.07.014. PMID: 31890511; PMCID: PMC6926308. | No rotator cuff |
| Mirzayan R, Andelman SM, Sethi PM, Baldino JB, Comer BJ, Obopilwe E, Morikawa D, Otto A, Mehl J, Murphy M, Mazzocca AD. Acellular dermal matrix augmentation significantly increases ultimate load to failure of pectoralis major tendon repair: a biomechanical study. J Shoulder Elbow Surg. 2020 Apr;29(4):728-735. doi: 10.1016/j.jse.2019.09.020. Epub 2019 Dec 16. PMID: 31859037. | Cadaveric study (no preclinical models) |
| Scheiderer B, Kia C, Obopilwe E, Johnson JD, Cote MP, Imhoff FB, Dyrna F, Beitzel K, Imhoff AB, Adams CR, Mazzocca AD, Morikawa D. Biomechanical Effect of Superior Capsule Reconstruction Using a 3-mm and 6-mm Thick Acellular Dermal Allograft in a Dynamic Shoulder Model. Arthroscopy. 2020 Feb;36(2):355-364. doi: 10.1016/j.arthro.2019.08.026. Epub 2019 Nov 29. PMID: 31791890. | Cadaveric study (no preclinical models) |
| Curtis DM, Lee CS, Qin C, Edgington J, Parekh A, Miller J, Tokish JM, Amirouche F, Athiviraham A. Superior Capsule Reconstruction With Subacromial Allograft Spacer: Biomechanical Cadaveric Study of Subacromial Contact Pressure and Superior Humeral Head Translation. Arthroscopy. 2020 Mar;36(3):680-686. doi: 10.1016/j.arthro.2019.09.047. Epub 2019 Nov 29. PMID: 31791889. | Cadaveric study (no preclinical models) |
| Lubowitz JH, Brand JC, Rossi MJ. Shoulder Superior Capsular Reconstruction Using Acellular Human Dermal Allograft. Arthroscopy. 2019 Oct;35(10):2769-2770. doi: 10.1016/j.arthro.2019.07.014. PMID: 31604489. | Editorial commentary |
| Tang X, Daneshmandi L, Awale G, Nair LS, Laurencin CT. Skeletal Muscle Regenerative Engineering. Regen Eng Transl Med. 2019 Sep;5(3):233-251. doi: 10.1007/s40883-019-00102-9. Epub 2019 Apr 2. PMID: 33778155; PMCID: PMC7992466. | Review |
| Li L, He WT, Qin BG, Liu XL, Yang JT, Gu LQ. Comparison between direct repair and human acellular nerve allografting during contralateral C7 transfer to the upper trunk for restoration of shoulder abduction and elbow flexion. Neural Regen Res. 2019 Dec;14(12):2132-2140. doi: 10.4103/1673-5374.262600. PMID: 31397352; PMCID: PMC6788224. | No rotator cuff |
| Mihata T. Editorial Commentary: Superior Capsule Reconstruction: Grafts for Superior Capsular Reconstruction Must Be Thick and Stiff. Arthroscopy. 2019 Aug;35(8):2535-2536. doi: 10.1016/j.arthro.2019.04.019. PMID: 31395197. | Editorial commentary |
| Zastrow RK, London DA, Parsons BO, Cagle PJ. Superior Capsule Reconstruction for Irreparable Rotator Cuff Tears: A Systematic Review. Arthroscopy. 2019 Aug;35(8):2525-2534.e1. doi: 10.1016/j.arthro.2019.02.053. PMID: 31395196. | Review |
| Li L, Yang J, Qin B, Wang H, Yang Y, Fang J, Chen G, Liu X, Tu Z, Gu L. Analysis of human acellular nerve allograft combined with contralateral C7 nerve root transfer for restoration of shoulder abduction and elbow flexion in brachial plexus injury: a mean 4-year follow-up. J Neurosurg. 2019 Apr 26;132(6):1914-1924. doi: 10.3171/2019.2.JNS182620. PMID: 31026835. | No rotator cuff |
| Martinez RA, Liston J, Archual AJ, Gui J, Drake DB, DeGeorge BR Jr. Digital Pulley Reconstruction Using Pulley Allografts: A Comparison With Traditional Tendon-Based Techniques. Ann Plast Surg. 2019 Jun;82(6S Suppl 5):S386-S388. doi: 10.1097/SAP.0000000000001793. PMID: 30870174. | No rotator cuff |
| Pennington WT. Author Reply to "Regarding 'Arthroscopic Superior Capsular Reconstruction With Acellular Dermal Allograft for the Treatment of Massive Irreparable Rotator Cuff Tears'". Arthroscopy. 2019 Jan;35(1):11. doi: 10.1016/j.arthro.2018.09.001. PMID: 30611336. | Editorial commentary |
| Narvani AA. Regarding "Arthroscopic Superior Capsular Reconstruction With Acellular Dermal Allograft for the Treatment of Massive Irreparable Rotator Cuff Tears". Arthroscopy. 2019 Jan;35(1):10-11. doi: 10.1016/j.arthro.2018.09.002. PMID: 30611334. | Letter to editor |
| Schaeffer CV, Dassoulas KR, Thuman J, Campbell CA. Early Functional Outcomes After Prepectoral Breast Reconstruction: A Case-Matched Cohort Study. Ann Plast Surg. 2019 Jun;82(6S Suppl 5):S399-S403. doi: 10.1097/SAP.0000000000001669. PMID: 30570559. | No rotator cuff |
| Makovicka JL, Patel KA, Tokish JM. Superior Capsular Reconstruction With the Addition of an Acromial Acellular Dermal Allograft Spacer. Arthrosc Tech. 2018 Oct 22;7(11):e1181-e1190. doi: 10.1016/j.eats.2018.08.003. PMID: 30533367; PMCID: PMC6261734. | Technical note |
| Laskovski JR, Boyd JA, Peterson EE, Abrams JS. Simplified Technique for Superior Capsular Reconstruction Using an Acellular Dermal Allograft. Arthrosc Tech. 2018 Oct 1;7(11):e1089-e1095. doi: 10.1016/j.eats.2018.07.002. PMID: 30533353; PMCID: PMC6261931. | Technical note |
| Liu Q, Yu Y, Reisdorf RL, Qi J, Lu CK, Berglund LJ, Amadio PC, Moran SL, Steinmann SP, An KN, Gingery A, Zhao C. Engineered tendon-fibrocartilage-bone composite and bone marrow-derived mesenchymal stem cell sheet augmentation promotes rotator cuff healing in a non-weight-bearing canine model. Biomaterials. 2019 Feb;192:189-198. doi: 10.1016/j.biomaterials.2018.10.037. Epub 2018 Oct 29. PMID: 30453215. | No decellularized patches |
| Greenall G, Carr A, Beard D, Rees J, Rangan A, Merritt N, Dritsaki M, Nagra NS, Baldwin M, Hopewell S, Cook JA. Systematic review of the surgical management of rotator cuff repair with an augmentative patch: a feasibility study protocol. Syst Rev. 2018 Nov 13;7(1):187. doi: 10.1186/s13643-018-0851-1. PMID: 30424809; PMCID: PMC6234662. | Review |
| Liu GM, Pan J, Zhang Y, Ning LJ, Luo JC, Huang FG, Qin TW. Bridging Repair of Large Rotator Cuff Tears Using a Multilayer Decellularized Tendon Slices Graft in a Rabbit Model. Arthroscopy. 2018 Sep;34(9):2569-2578. doi: 10.1016/j.arthro.2018.04.019. Epub 2018 Aug 2. PMID: 30078689. | No decellularized patches |
| Kenney RJ, Mannava S, Maloney MD. Allograft Use in Shoulder Surgery: Instability and Rotator Cuff. Sports Med Arthrosc Rev. 2018 Sep;26(3):145-148. doi: 10.1097/JSA.0000000000000208. PMID: 30059450. | Review |
| Laskovski J, Urchek R. Endoscopic Gluteus Medius and Minimus Repair With Allograft Augmentation Using Acellular Human Dermis. Arthrosc Tech. 2018 Feb 12;7(3):e225-e230. doi: 10.1016/j.eats.2017.08.073. PMID: 29876243; PMCID: PMC5989483. | No rotator cuff |
| Liu Q, Hatta T, Qi J, Liu H, Thoreson AR, Amadio PC, Moran SL, Steinmann SP, Gingery A, Zhao C. Novel engineered tendon-fibrocartilage-bone composite with cyclic tension for rotator cuff repair. J Tissue Eng Regen Med. 2018 Jul;12(7):1690-1701. doi: 10.1002/term.2696. Epub 2018 Jun 3. PMID: 29763517. | No preclinical models |
| Elhassan BT, Cox RM, Shukla DR, Lee J, Murthi AM, Tashjian RZ, Abboud JA. Management of Failed Rotator Cuff Repair in Young Patients. Instr Course Lect. 2018 Feb 15;67:143-154. PMID: 31411408. | Full text not available |
| Milks RA, Kolmodin JD, Ricchetti ET, Iannotti JP, Derwin KA. Augmentation with a reinforced acellular fascia lata strip graft limits cyclic gapping of supraspinatus repairs in a human cadaveric model. J Shoulder Elbow Surg. 2018 Jun;27(6):1105-1111. doi: 10.1016/j.jse.2017.12.016. Epub 2018 Feb 3. PMID: 29398399. | Cadaveric study (no preclinical models) |
| Logli AL, Twu J, Bear BJ, Lindquist JR, Schoenfeldt TL, Korcek KJ. Arthroscopic Partial Trapeziectomy With Soft Tissue Interposition for Symptomatic Trapeziometacarpal Arthritis: 6-Month and 5-Year Minimum Follow-Up. J Hand Surg Am. 2018 Apr;43(4):384.e1-384.e7. doi: 10.1016/j.jhsa.2017.10.016. Epub 2017 Nov 11. PMID: 29132788. | No rotator cuff |
| Distal Biceps Repair With Acellular Dermal Graft Augmentation, Operative Techniques in Sports Medicine, Volume 26, Issue 2, 2018, Pages 130-135, ISSN 1060-1872,   - https://doi.org/10.1053/j.otsm.2018.02.013. | No rotator cuff |
| Li, H., Zhou, B. and Tang, K. (2021), Advancement in Arthroscopic Superior Capsular Reconstruction for Irreparable Massive Rotator Cuff Tear. Orthop Surg, 13: 1951-1959. https://doi.org/10.1111/os.12976 | Review |
| Cole W, Samsell B, Moore MA. Achilles Tendon Augmented Repair Using Human Acellular Dermal Matrix: A Case Series. J Foot Ankle Surg. 2018 Nov-Dec;57(6):1225-1229. doi: 10.1053/j.jfas.2018.03.006. Epub 2018 May 18. PMID: 29779989. | No rotator cuff |
| Stone, Michael A. MD*; Namdari, Surena MD†; Omid, Reza MD*. Superior Capsule Reconstruction for Irreparable Rotator Cuff Tears: A Novel Technique Using Mulberry Knots. Techniques in Orthopaedics 33(3):p e9-e12, September 2018. \| DOI: 10.1097/BTO.0000000000000232 | Technical note |
| Sunwoo JY, Murrell GAC. Interposition Graft Repair of Irreparable Rotator Cuff Tears: A Review of Biomechanics and Clinical Outcomes. J Am Acad Orthop Surg. 2020 Oct 1;28(19):e829-e838. doi: 10.5435/JAAOS-D-19-00500. PMID: 32649438. | Review |
| Quigley R, Verma N, Evuarherhe A Jr, Cole BJ. Rotator Cuff Repair with Graft Augmentation Improves Function, Decreases Revisions, and Is Cost-Effective. Arthroscopy. 2022 Jul;38(7):2166-2174. doi: 10.1016/j.arthro.2022.01.011. Epub 2022 Jan 20. PMID: 35066111. | No preclinical models |
| Galvin JW, Kenney R, Curry EJ, Parada SA, Eichinger JK, Voloshin I, Li X. Superior Capsular Reconstruction for Massive Rotator Cuff Tears: A Critical Analysis Review. JBJS Rev. 2019 Jun;7(6):e1. doi: 10.2106/JBJS.RVW.18.00072. PMID: 31166218. | Review |
| Lu, H., Tang, Y., Liu, F., Xie, S., Qu, J. and Chen, C. (2019), Comparative Evaluation of the Book-Type Acellular Bone Scaffold and Fibrocartilage Scaffold for Bone-Tendon Healing. J. Orthop. Res., 37: 1709-1722. https://doi.org/10.1002/jor.24301 | No rotator cuff |
| Xiping Jiang, Yunfan Kong, Mitchell Kuss, Joel Weisenburger, Hani Haider, Robert Harms, Wen Shi, Bo Liu, Wen Xue, Jianghu Dong, Jingwei Xie, Philipp Streubel, Bin Duan, 3D bioprinting of multilayered scaffolds with spatially differentiated ADMSCs for rotator cuff tendon-to-bone interface regeneration, Applied Materials Today, Volume 27, 2022, 101510, ISSN 2352-9407, https://doi.org/10.1016/j.apmt.2022.101510. | No decellularized patches |
| Al-Ani Z, Monga P, Walton M, Funk L, Basu S. An OrthoRadiological review of superior capsularreconstruction in the shoulder. Skeletal Radiol. 2021 Feb;50(2):267-280. doi: 10.1007/s00256-020-03577-8. Epub 2020 Aug 13. PMID: 32789679. | Review |
| Shim IK, Kang MS, Lee ES, Choi JH, Lee YN, Koh KH. Decellularized Bovine Pericardial Patch Loaded With Mesenchymal Stromal Cells Enhance the Mechanical Strength and Biological Healing of Large-to-Massive Rotator Cuff Tear in a Rat Model. Arthroscopy. 2022 Nov;38(11):2987-3000. doi: 10.1016/j.arthro.2022.06.004. Epub 2022 Jun 16. PMID: 35716989. | Full text not available |
| Cui J, Ning LJ, Yao X, Zhang Y, Zhang YJ, He SK, Zhang Z, Ding W, Luo JC, Qin TW. Influence of the integrity of tendinous membrane and fascicle on biomechanical characteristics of tendon-derived scaffolds. Biomed Mater. 2020 Dec 23;16(1):015029. doi: 10.1088/1748-605X/abc203. PMID: 33065568. | No preclinical models |
| Baojun Chen, Yongping Liang, Lang Bai, Meiguang Xu, Jing Zhang, Baolin Guo, Zhanhai Yin,Sustained release of magnesium ions mediated by injectable self-healing adhesive hydrogel promotes fibrocartilaginous interface regeneration in the rabbit rotator cuff tear model, Chemical Engineering Journal,   - Volume 396, 2020, 125335, ISSN 1385-8947, - https://doi.org/10.1016/j.cej.2020.125335. | No decellularized patches |
| Melandri D, Marongiu F, Carboni A, Rubino C, Razzano S, Purpura V, Minghetti P, Bondioli E. A New Human-Derived Acellular Dermal Matrix for 1-Stage Coverage of Exposed Tendons in the Foot. Int J Low Extrem Wounds. 2020 Mar;19(1):78-85. doi: 10.1177/1534734619884422. Epub 2019 Nov 4. PMID: 31679415. | No rotator cuff |
| Gouk, Conor J.C. MSc, MBChB; Shulman, Ryan M. MBBS, FRANZCR; Buchan, Craig MBBS (Hons), FRANZCR; Taylor, Fraser J. BSc, MBChB, FRACS. Chronic Distal Triceps Rupture Repaired Using Acellular Dermal Allograft as an Interposition Graft. Techniques in Shoulder & Elbow Surgery 20(1):p 35-38, March 2019. \| DOI: 10.1097/BTE.0000000000000161 | No rotator cuff |
| Capella-Monsonís H, Zeugolis DI. Decellularized xenografts in regenerative medicine: From processing to clinical application. Xenotransplantation. 2021 Jul;28(4):e12683. doi: 10.1111/xen.12683. Epub 2021 Mar 12. PMID: 33709410. | Review |
| Tang Y, Chen C, Liu F, Xie S, Qu J, Li M, Li Z, Li X, Shi Q, Li S, Li X, Hu J, Lu H. Structure and ingredient-based biomimetic scaffolds combining with autologous bone marrow-derived mesenchymal stem cell sheets for bone-tendon healing. Biomaterials. 2020 May;241:119837. doi: 10.1016/j.biomaterials.2020.119837. Epub 2020 Feb 18. PMID: 32109704. | No rotator cuff |
| Zhou Y, Xie S, Tang Y, Li X, Cao Y, Hu J, Lu H. Effect of book-shaped acellular tendon scaffold with bone marrow mesenchymal stem cells sheets on bone-tendon interface healing. J Orthop Translat. 2020 Mar 24;26:162-170. doi: 10.1016/j.jot.2020.02.013. PMID: 33437635; PMCID: PMC7773951. | No rotator cuff |
| Sarrafian TL, Bodine SC, Murphy B, Grayson JK, Stover SM. Extracellular matrix scaffolds for treatment of large volume muscle injuries: A review. Vet Surg. 2018 May;47(4):524-535. doi: 10.1111/vsu.12787. Epub 2018 Mar 30. PMID: 29603757. | Review |
| Frank RM, Cvetanovich G, Savin D, Romeo AA. Superior Capsular Reconstruction: Indications, Techniques, and Clinical Outcomes. JBJS Rev. 2018 Jul;6(7):e10. doi: 10.2106/JBJS.RVW.17.00141. PMID: 30063502. Frank RM, Cvetanovich G, Savin D, Romeo AA. Superior Capsular Reconstruction: Indications, Techniques, and Clinical Outcomes. JBJS Rev. 2018 Jul;6(7):e10. doi: 10.2106/JBJS.RVW.17.00141. PMID: 30063502. | Review |
| Cramer MC, Badylak SF.  Extracellular Matrix-Based Biomaterials and Their Influence Upon Cell Behavior. Ann Biomed Eng. 2020 Jul;48(7):2132-2153. doi: 10.1007/s10439-019-02408-9. Epub 2019 Nov 18. PMID: 31741227; PMCID: PMC7231673. | Review |
| Cui J, Ning LJ, Wu FP, Hu RN, Li X, He SK, Zhang YJ, Luo JJ, Luo JC, Qin TW. Biomechanically and biochemically functional scaffold for recruitment of endogenous stem cells to promote tendon regeneration. NPJ Regen Med. 2022 Apr 26;7(1):26. doi: 10.1038/s41536-022-00220-z. PMID: 35474221; PMCID: PMC9043181. | No rotator cuff |
| Lei T, Zhang T, Ju W, Chen X, Heng BC, Shen W, Yin Z. Biomimetic strategies for tendon/ligament-to-bone interface regeneration. Bioact Mater. 2021 Feb 2;6(8):2491-2510. doi: 10.1016/j.bioactmat.2021.01.022. PMID: 33665493; PMCID: PMC7889437. | Review |
| Tuleubayev BE, Anapiya BB, Kurmangaliyev YT, Abugaliyev KR. Successful Treatment Outcomes for Partial Thickness Burns by Innovative Bovine Peritoneum Dressing. Plast Reconstr Surg Glob Open. 2022 Feb 24;10(2):e4150. doi: 10.1097/GOX.0000000000004150. PMID: 35233339; PMCID: PMC8878626. | No rotator cuff |
| Shengnan Q, Bennett S, Wen W, Aiguo L, Jiake X. The role of tendon derived stem/progenitor cells and extracellular matrix components in the bone tendon junction repair. Bone. 2021 Dec;153:116172. doi: 10.1016/j.bone.2021.116172. Epub 2021 Sep 14. PMID: 34506992. | Review |
| Alap U. Patel, Serena J. Day, Megan Pencek, Lauren O. Roussel, Jose G. Christiano, Imran R. Punekar, Peter F. Koltz, Howard N. Langstein,   - Functional return after implant-based breast reconstruction: A prospective study of objective and patient-reported outcomes, Journal of Plastic, Reconstructive & Aesthetic Surgery, Volume 73, Issue 5, 2020, Pages 850-855, ISSN 1748-6815, - https://doi.org/10.1016/j.bjps.2019.11.038. | No rotator cuff |
| Samson DJ, Gachabayov M, Latifi R. Biologic Mesh in Surgery: A Comprehensive Review and Meta-Analysis of Selected Outcomes in 51 Studies and 6079 Patients. World J Surg. 2021 Dec;45(12):3524-3540. doi: 10.1007/s00268-020-05887-3. Epub 2021 Jan 8. PMID: 33416939. | Review |
| The biology and biomechanics of grafts and implants Barber, F. Alan | Book chapter |
| Nedal Al-Khatib, Ivan Wong, 27 - Bridging reconstruction for massive rotator cuff tears, Brian J. Cole, Jorge Chahla, Ron Gilat | Book chapter |
| Tibone JE, Mansfield C, Kantor A, Giordano J, Lin CC, Itami Y, McGarry MH, Adamson GJ, Lee TQ. Human Dermal Allograft Superior Capsule Reconstruction With Graft Length Determined at Glenohumeral Abduction Angles of 20° and 40° Decreases Joint Translation and Subacromial Pressure Without Compromising Range of Motion: A Cadaveric Biomechanical Study. Arthroscopy. 2022 May;38(5):1398-1407. doi: 10.1016/j.arthro.2021.11.007. Epub 2021 Nov 14. PMID: 34785299. | Cadaveric study (no preclinical models) |
| Tognetti L, Pianigiani E, Ierardi F, Lorenzini G, Casella D, Liso FG, De Pascalis A, Cinotti E, Rubegni P. The use of human acellular dermal matrices in advanced wound healing and surgical procedures: State of the art. Dermatol Ther. 2021 Jul;34(4):e14987. doi: 10.1111/dth.14987. Epub 2021 May 24. PMID: 33993627. | Review |
| Osama R. Aldhafian, Kyung-Ho Choi, Han-Suk Cho, Fahad Alarishi, Yang-Soo Kim, Outcome of intraoperative injection of collagen in arthroscopic repair of full-thickness rotator cuff tear: a retrospective cohort study, Journal of Shoulder and Elbow Surgery, Volume 32, Issue 9, 2023, Pages e429-e436, ISSN 1058-2746,   - https://doi.org/10.1016/j.jse.2023.03.002. | Clinical study |
| Mirzayan R. Preliminary Outcomes of Arthroscopic Biologic Tuberoplasty in the Treatment of Massive Irreparable Rotator Cuff Tears. Cureus. 2023 Jan 30;15(1):e34402. doi: 10.7759/cureus.34402. PMID: 36733564; PMCID: PMC9887923. | Technical note |
| Gbejuade H, Patel MS, Singh H, Modi A. Reconstruction of irreparable rotator cuff tears with an acellular dermal matrix in elderly patients without joint arthritis. Shoulder Elbow. 2022 Jul;14(1 Suppl):83-89. doi: 10.1177/1758573220965535. Epub 2020 Oct 15. PMID: 35845625; PMCID: PMC9284257. | Clinical study |
| Cha EDK, Shultz K, Chan K, Choi J. Longitudinal efficacy of acellular dermal allograft following superior capsular reconstruction of irreparable rotator cuff tears. J Orthop. 2022 Jun 23;33:31-36. doi: 10.1016/j.jor.2022.06.012. PMID: 35801200; PMCID: PMC9253897. | Clinical study |
| Lee GW, Kim JY, Lee HW, Yoon JH, Noh KC. Clinical and Anatomical Outcomes of Arthroscopic Repair of Large Rotator Cuff Tears with Allograft Patch Augmentation: A Prospective, Single-Blinded, Randomized Controlled Trial with a Long-term Follow-up. Clin Orthop Surg. 2022 Jun;14(2):263-271. doi: 10.4055/cios21135. Epub 2022 May 13. PMID: 35685982; PMCID: PMC9152903. | Clinical study |
| Choi S, Kim G, Lee Y, Kim BG, Jang I, Kim JH. Patch augmentation does not provide better clinical outcomes than arthroscopic rotator cuff repair for large to massive rotator cuff tears. Knee Surg Sports Traumatol Arthrosc. 2022 Nov;30(11):3851-3861. doi: 10.1007/s00167-022-06975-8. Epub 2022 May 6. PMID: 35522311. | Clinical study |
| Ning LJ, Cui J, He SK, Hu RN, Yao X, Zhang Y, Ding W, Zhang YJ, Luo JC, Qin TW. Constructing a highly bioactive tendon-regenerative scaffold by surface modification of tissue-specific stem cell-derived extracellular matrix. Regen Biomater. 2022 Apr 20;9:rbac020. doi: 10.1093/rb/rbac020. PMID: 35480863; PMCID: PMC9036902. | *In vitro* study |
| Cromheecke M, Garret J, Deranlot J, Bonnevialle N, Gaudin S, Lädermann A, Nourissat G; French Arthroscopic Society (SFA). Low healing rates and moderate functional outcome after arthroscopic superior capsular reconstruction using a porcine xenograft. Knee Surg Sports Traumatol Arthrosc. 2022 Jul;30(7):2528-2534. doi: 10.1007/s00167-022-06916-5. Epub 2022 Feb 23. PMID: 35199186. | Clinical study |
| Garofalo R, Fontanarosa A, De Crescenzo A, Conti M, Calbi R, Castagna A. Does arthroscopic superior capsule reconstruction using porcine dermal xenograft represent a viable option in case of massive irreparable posterosuperior rotator cuff tear? Arch Orthop Trauma Surg. 2023 Jan;143(1):439-445. doi: 10.1007/s00402-022-04335-2. Epub 2022 Jan 27. PMID: 35084550. | Clinical study |
| Shin SJ, Lee S, Hwang JY, Lee W, Koh KH. Tear pattern after superior capsular reconstruction using an acellular dermal matrix allograft. J Shoulder Elbow Surg. 2022 Jun;31(6):e279-e288. doi: 10.1016/j.jse.2021.12.009. Epub 2022 Jan 10. PMID: 35026395. | Clinical study |
| Modi A, Haque A, Deore V, Singh HP, Pandey R. Interposition GraftJacket allografts for irreparable rotator cuff tears. Bone Joint J. 2022 Jan;104-B(1):91-96. doi: 10.1302/0301-620X.104B1.BJJ-2021-0826.R1. PMID: 34969271. | Clinical study |
| Evuarherhe A Jr, Condron NB, Gilat R, Knapik DM, Patel S, Wagner KR, Garrigues GE, Romeo A, Verma N, Cole BJ. Defining Clinically Significant Outcomes Following Superior Capsular Reconstruction With Acellular Dermal Allograft. Arthroscopy. 2022 May;38(5):1444-1453.e1. doi: 10.1016/j.arthro.2021.11.039. Epub 2021 Dec 2. PMID: 34863902. | Clinical study |
| Shin SJ, Lee S, Hwang JY, Lee W, Koh KH. Superior Capsular Reconstruction Using Acellular Dermal Allograft Combined With Remaining Rotator Cuff Augmentation Improved Shoulder Pain and Function at 1 Year After The Surgery. Arthroscopy. 2022 Apr;38(4):1089-1098. doi: 10.1016/j.arthro.2021.10.027. Epub 2021 Nov 10. PMID: 34767952. | Clinical study |
| Cable BM, Farooqi AS, Tsai S, Plyler R, Lee A, Parisien RL, Kelly JD 4th. Humeral Head Morphology Influences Outcomes of Arthroscopic Interposition Glenoid Patch Allograft for Glenohumeral Arthritis. Arthrosc Sports Med Rehabil. 2021 Aug 14;3(5):e1421-e1429. doi: 10.1016/j.asmr.2021.07.004. PMID: 34712980; PMCID: PMC8527262. | Clinical study |
| Lädermann A, Denard PJ, Barth J, Bonnevialle N, Lejeune E; SCRSFA group; Bothorel H; Francophone Arthroscopy Society (SFA); Nourrissat G. Superior capsular reconstruction for irreparable rotator cuff tears: Autografts versus allografts. Orthop Traumatol Surg Res. 2021 Dec;107(8S):103059. doi: 10.1016/j.otsr.2021.103059. Epub 2021 Sep 16. PMID: 34537391. | Clinical study |
| Awad MA, Sparavalo S, Ma J, King JP, Wong I. Interposition Graft Bridging Reconstruction of Irreparable Rotator Cuff Tears Using Acellular Dermal Matrix: Medium-Term Results. Arthroscopy. 2022 Mar;38(3):692-698. doi: 10.1016/j.arthro.2021.08.001. Epub 2021 Aug 9. PMID: 34384854. | Clinical study |
| Namdari S, Nicholson T, Brolin TJ, Lu J, Abboud JA, Lazarus MD. Healing and Functional Results of Dermal Allograft Augmentation of Complex and Revision Rotator Cuff Repairs. Am J Sports Med. 2021 Jul;49(8):2042-2047. doi: 10.1177/03635465211015194. Epub 2021 May 20. PMID: 34015245. | Clinical study |
| Lederman ES, McLean JB, Bormann KT, Guttmann D, Ortega KD, Miles JW, Hartzler RU, Dorfman AL, Softic D, Qin X. Histologic case series of human acellular dermal matrix in superior capsule reconstruction. J Shoulder Elbow Surg. 2021 Sep;30(9):2146-2155. doi: 10.1016/j.jse.2021.01.019. Epub 2021 Feb 16. PMID: 33600900. | Clinical study |
| Bernstein DT, Kluemper CT, Kearns KA. Anterior Capsular Reconstruction with Dermal Allograft in Combination With Anatomic Glenohumeral Arthroplasty: A Case Report. JBJS Case Connect. 2020 Dec 24;10(4):e20.00214. doi: 10.2106/JBJS.CC.20.00214. PMID: 33449549. | Clinical study |
| Goldenberg BT, Lacheta L, Dekker TJ, Spratt JD, Nolte PC, Millett PJ. Biologics to Improve Healing in Large and Massive Rotator Cuff Tears: A Critical Review. Orthop Res Rev. 2020 Oct 13;12:151-160. doi: 10.2147/ORR.S260657. PMID: 33116954; PMCID: PMC7568683. | Clinical study |
| Gilat R, Haunschild ED, Williams BT, Fu MC, Garrigues GE, Romeo AA, Verma NN, Cole BJ. Patient Factors Associated With Clinical Failure Following Arthroscopic Superior Capsular Reconstruction. Arthroscopy. 2021 Feb;37(2):460-467. doi: 10.1016/j.arthro.2020.09.038. Epub 2020 Sep 28. PMID: 32998042. | Clinical study |
| Ferrando A, Kingston R, Delaney RA. Superior capsular reconstruction using a porcine dermal xenograft for irreparable rotator cuff tears: outcomes at minimum two-year follow-up. J Shoulder Elbow Surg. 2021 May;30(5):1053-1059. doi: 10.1016/j.jse.2020.08.020. Epub 2020 Sep 2. PMID: 32890682. | Clinical study |
| Hall T, Danielson K, Brandenburg S, Matelic T. A case series of recurrent myotendinous rotator cuff tears repaired and augmented with dermal allograft: clinical outcomes at two years. J Shoulder Elbow Surg. 2020 Nov;29(11):2264-2271. doi: 10.1016/j.jse.2020.03.021. Epub 2020 Jun 9. PMID: 32741564. | Clinical study |
| Makki D, Tang QO, Sandher D, Morgan BW, Ravenscroft M. Arthroscopic Superior Capsular Reconstruction of the Shoulder Using Dermal Allograft. Orthopedics. 2020 Jul 1;43(4):215-220. doi: 10.3928/01477447-20200428-05. Epub 2020 May 7. PMID: 32379339. | Clinical study |
| Lacheta L, Horan MP, Schairer WW, Goldenberg BT, Dornan GJ, Pogorzelski J, Millett PJ. Clinical and Imaging Outcomes After Arthroscopic Superior Capsule Reconstruction With Human Dermal Allograft for Irreparable Posterosuperior Rotator Cuff Tears: A Minimum 2-Year Follow-Up. Arthroscopy. 2020 Apr;36(4):1011-1019. doi: 10.1016/j.arthro.2019.12.024. Epub 2020 Jan 15. PMID: 31953193. | Clinical study |
| Burkhart SS, Pranckun JJ, Hartzler RU. Superior Capsular Reconstruction for the Operatively Irreparable Rotator Cuff Tear: Clinical Outcomes Are Maintained 2 Years After Surgery. Arthroscopy. 2020 Feb;36(2):373-380. doi: 10.1016/j.arthro.2019.08.035. Epub 2019 Dec 18. PMID: 31864817. | Clinical study |
| Gracitelli MEC, Beraldo RA, Malavolta EA, Assunção JH, Oliveira DRO, Ferreira Neto AA. Superior Capsular Reconstruction with Fascia Lata Allograft for Irreparable Supraspinatus Tendon Tears. Rev Bras Ortop (Sao Paulo). 2019 Sep;54(5):591-596. doi: 10.1016/j.rbo.2017.11.011. Epub 2019 Oct 9. PMID: 31686714; PMCID: PMC6819162. | Clinical study |
| Altintas B, Scibetta AC, Storaci HW, Lacheta L, Anderson NL, Millett PJ. Biomechanical and Histopathological Analysis of a Retrieved Dermal Allograft After Superior Capsule Reconstruction: A Case Report. Arthroscopy. 2019 Oct;35(10):2959-2965. doi: 10.1016/j.arthro.2019.07.006. PMID: 31604518. | Clinical study |
| Hartzler RU, Softic D, Qin X, Dorfman A, Adams CR, Burkhart SS. The Histology of a Healed Superior Capsular Reconstruction Dermal Allograft: A Case Report. Arthroscopy. 2019 Oct;35(10):2950-2958. doi: 10.1016/j.arthro.2019.06.024. PMID: 31604517. | Clinical study |
| Samade R, Jones GL, Bishop JY. Evaluation of an Incorporated Superior Capsular Reconstruction Graft: A Case Report. JBJS Case Connect. 2019 Jul-Sep;9(3):e0378. doi: 10.2106/JBJS.CC.18.00378. PMID: 31584901. | Clinical study |
| Morgan B, Manning CJ, Sandher D, Ravenscroft M. Acromioclavicular joint biological resurfacing using ArthroFLEX® graft. Ann R Coll Surg Engl. 2020 Feb;102(2):161. doi: 10.1308/rcsann.2019.0133. Epub 2019 Sep 20. PMID: 31537112; PMCID: PMC6996422. | Technical note |
| Mirzayan R, Stone MA, Batech M, Acevedo DC, Singh A. Failed Dermal Allograft Procedures for Irreparable Rotator Cuff Tears Can Still Improve Pain and Function: The "Biologic Tuberoplasty Effect". Orthop J Sports Med. 2019 Aug 20;7(8):2325967119863432. doi: 10.1177/2325967119863432. PMID: 31457066; PMCID: PMC6702775. | Clinical study |
| Gouk CJC, Shulman RM, Buchan C, Thomas MJE, Taylor FJ. Failure of Dermal Allograft Repair of Massive Rotator Cuff Tears in Magnetic Resonance Imaging and Clinical Assessment. Clin Orthop Surg. 2019 Jun;11(2):200-207. doi: 10.4055/cios.2019.11.2.200. Epub 2019 May 9. PMID: 31156773; PMCID: PMC6526136. | Clinical study |
| Ravenscroft MJ, Riley JA, Morgan BW, Sandher DS, Odak SS, Joseph P. Histological incorporation of acellular dermal matrix in the failed superior capsule reconstruction of the shoulder. J Exp Orthop. 2019 May 25;6(1):21. doi: 10.1186/s40634-019-0189-1. PMID: 31129749; PMCID: PMC6535153. | Clinical study |
| Hortensius RA, Ebens JH, Dewey MJ, Harley BAC. Incorporation of the Amniotic Membrane as an Immunomodulatory Design Element in Collagen Scaffolds for Tendon Repair. ACS Biomater Sci Eng. 2018 Dec 10;4(12):4367-4377. doi: 10.1021/acsbiomaterials.8b01154. Epub 2018 Oct 19. PMID: 30693317; PMCID: PMC6347397. | *In vitro* study |
| Maillot C, Harly E, Demezon H, Le Huec JC. Surgical repair of large-to-massive rotator cuff tears seems to be a better option than patch augmentation or débridement and biceps tenotomy: a prospective comparative study. J Shoulder Elbow Surg. 2018 Sep;27(9):1545-1552. doi: 10.1016/j.jse.2018.05.023. Epub 2018 Jul 3. PMID: 29980338. | Clinical study |
| Pennington WT, Bartz BA, Pauli JM, Walker CE, Schmidt W. Arthroscopic Superior Capsular Reconstruction With Acellular Dermal Allograft for the Treatment of Massive Irreparable Rotator Cuff Tears: Short-Term Clinical Outcomes and the Radiographic Parameter of Superior Capsular Distance. Arthroscopy. 2018 Jun;34(6):1764-1773. doi: 10.1016/j.arthro.2018.01.009. Epub 2018 Feb 15. PMID: 29456069. | Clinical study |
| Hohn EA, Gillette BP, Burns JP. Outcomes of arthroscopic revision rotator cuff repair with acellular human dermal matrix allograft augmentation. J Shoulder Elbow Surg. 2018 May;27(5):816-823. doi: 10.1016/j.jse.2017.09.026. Epub 2017 Dec 1. PMID: 29198938. | Clinical study |
| Snow M, Kuiper JH, James S, Keeling E, Rich S, Amit P. A pilot randomised controlled trial assessing standard versus dermal patch-augmented rotator cuff repair found no adverse effects and suggest future trials need a minimum of 150 patients. Knee Surg Sports Traumatol Arthrosc. 2023 Jul;31(7):2654-2661. doi: 10.1007/s00167-023-07356-5. Epub 2023 Mar 2. PMID: 36862197. | Clinical study |

**Table 2.** Raw data of SYRCLE’s risk of bias

| **Y**: Yes (Low risk of bias). -**U**: (Unclear). -**N:** (High risk of bias). | | |  |  |  |  |  |  |  |  |
| --- | --- | --- | --- | --- | --- | --- | --- | --- | --- | --- |
|  | **ITEMS** | | | | | | | | | |
|  | **Sequence generation** | **Baseline characteristics** | **Allocation concealment** | **Random housing** | **Blinding**  **(Performance)** | **Random outcome assessment** | **Blinding**  **(Detection)** | **Incomplete outcome data** | **Selective outcome reporting** | **Other sources of bias** |
| Thangarajah 2018 | **N** | **U** | **N** | **N** | **N** | **N** | **N** | **Y** | **Y** | **Y** |
| Shim 2022 | **N** | **Y** | **N** | **N** | **N** | **N** | **Y** | **Y** | **Y** | **Y** |
| Chen 2020 | **N** | **Y** | **N** | **N** | **N** | **N** | **N** | **Y** | **Y** | **Y** |
| Ide 2018 | **N** | **Y** | **N** | **N** | **N** | **N** | **Y** | **U** | **Y** | **Y** |
| De Lima Santos 2020 | **N** | **Y** | **N** | **N** | **N** | **N** | **U** | **Y** | **Y** | **Y** |
| De Lima Santos 2021 | **N** | **U** | **N** | **N** | **U** | **N** | **N** | **Y** | **Y** | **Y** |
| He 2021 | **N** | **Y** | **Y** | **N** | **N** | **N** | **N** | **U** | **Y** | **Y** |
| Yildiz 2018 | **N** | **U** | **N** | **N** | **N** | **N** | **Y** | **U** | **Y** | **Y** |
| Yuan 2022 | **N** | **Y** | **N** | **N** | **N** | **N** | **Y** | **Y** | **Y** | **Y** |
| Yuan 2022 | **N** | **U** | **N** | **N** | **N** | **N** | **N** | **Y** | **Y** | **Y** |
| Credille 2023 | **N** | **Y** | **N** | **N** | **N** | **N** | **U** | **Y** | **Y** | **U** |
| Smith 2020 | **N** | **Y** | **N** | **N** | **N** | **N** | **Y** | **Y** | **Y** | **U** |
| Chen 2022 | **N** | **Y** | **N** | **N** | **N** | **U** | **U** | **Y** | **Y** | **Y** |
